# Virus-like nanostructures for tuning immune response

**DOI:** 10.1038/srep16728

**Published:** 2015-11-18

**Authors:** Rashad Mammadov, Goksu Cinar, Nuray Gunduz, Melis Goktas, Handan Kayhan, Sehmus Tohumeken, Ahmet E. Topal, Ilghar Orujalipoor, Tuncay Delibasi, Aykutlu Dana, Semra Ide, Ayse B. Tekinay, Mustafa O. Guler

**Affiliations:** 1 Institute of Materials Science and Nanotechnology, National Nanotechnology Research Center (UNAM), Bilkent University, Ankara 06800, Turkey; 2 Adult Hematology Laboratory, School of Medicine, Gazi University, Ankara 06500, Turkey; 3 Department of Physics Engineering, Hacettepe University, 06800, Ankara, Turkey; 4 Hacettepe University and Diskapi Research and Training Hospital, 06800, Ankara, Turkey

## Abstract

Synthetic vaccines utilize viral signatures to trigger immune responses. Although the immune responses raised against the biochemical signatures of viruses are well characterized, the mechanism of how they affect immune response in the context of physical signatures is not well studied. In this work, we investigated the ability of zero- and one-dimensional self-assembled peptide nanostructures carrying unmethylated CpG motifs (signature of viral DNA) for tuning immune response. These nanostructures represent the two most common viral shapes, spheres and rods. The nanofibrous structures were found to direct immune response towards Th1 phenotype, which is responsible for acting against intracellular pathogens such as viruses, to a greater extent than nanospheres and CpG ODN alone. In addition, nanofibers exhibited enhanced uptake into dendritic cells compared to nanospheres or the ODN itself. The chemical stability of the ODN against nuclease-mediated degradation was also observed to be enhanced when complexed with the peptide nanostructures. *In vivo* studies showed that nanofibers promoted antigen-specific IgG production over 10-fold better than CpG ODN alone. To the best of our knowledge, this is the first report showing the modulation of the nature of an immune response through the shape of the carrier system.

The development of novel vaccines is an urgent issue due to the prospect of pandemic threats by infectious diseases such as flu (influenza virus) and Ebola. Current vaccinology mainly relies on inactivated or live attenuated viruses to introduce the characteristics of the virus to the immune system as much as possible^1^. Widely used egg-based vaccine production depends on viral growth characteristics in eggs. Slow-growing viruses or scarcity of egg resources might delay vaccine production during pandemics, when time is the limiting factor. Moreover, live attenuated viruses, which are superior to inactivated viruses in eliciting immune responses^1^,^2^, carry the risk of becoming virulent (as in the case of oral poliovirus vaccine^3^) or causing side effects in immuno-compromised individuals^4^. Due to the above-mentioned reasons, the rational design of safer vaccines with high effectiveness, and easy production processes is vitally needed. To achieve this purpose, the principles required to drive the immune response to the desired context (such as what type of cytokines and co-stimulatory molecules are expressed or what type of innate and adaptive immune cells are activated in response to a given stimulus) should be well understood^5^. These principles are based on the mechanisms by which immune cells recognize and respond to different features of pathogens, and especially to their biochemical and biophysical characteristics. In this context, pathogen-associated molecular patterns (PAMPs) stand out as biochemical pathogenic signatures, and can be used as adjuvants to enhance the immunogenicity of antigens^2^,^6^. PAMPs trigger the elevation of antigen presentation and cytokine secretion by innate immune cells, which eventually shape the adaptive immune response^7^,^8^. Among PAMPs, DNA with unmethylated CpG motifs, a signature of bacterial/viral DNAs, have been studied in great deal and demonstrated to boost humoral and cellular immune responses to vaccines^9^,^10^,^11^,^12^.

In nature, biochemical signals act in the context of biophysical features, such as the size and shape of a pathogen. This synergy plays a critical role in shaping the immune response and should be projected onto the vaccine for robust effectiveness. For example, the physical proximity of the antigen and adjuvant (*e.g.* CpG DNA), which allows both to be internalized by the same immune cells, has been shown to be critical for inducing a strong immune response^10^,^13^. The organization of molecular structures on the pathogens is another physical factor influencing immune response^14^. In this context, the density of antigenic epitopes has been shown to affect the IgG responses of B-cells^15^. In addition, repetitive patterns on the pathogen surface, which are especially prevalent in viruses, facilitate antigen processing and induce strong signaling in B-cells by cross-linking their receptors^16^. Size also affects the immune response raised against antigens and adjuvants. In order to reach lymphoid organs, antigens should enter the lymphatic system, which appears to happen more efficiently with particle sizes in the 20–200 nm range^16^. The size of CpG oligodeoxynucleotide (ODN)-uploaded particles were shown to affect the quantity of cytotoxic T-cells generated against the antigen^17^. Particulate antigens have been shown to be superior to soluble antigens in reaching major histocompatibility complex I (MHCI) pathway for cross-presentation^18^. A wide range of materials, including cationic microparticles, liposomes/virosomes, virus-like particles, nanoparticles and nanorods have been proposed for making antigen-adjuvant complexes particulate, for their delivery in close proximity, and for adjusting the size and shape of the complex^16^,^19^. They boost the immune reaction to antigen/adjuvant through enhanced interaction with cells, cellular uptake and protection from enzymatic degradation^16^,^19^. Nanoparticle carriers can also alter the nature of the immune response (*e.g.* inducing different cytokine profile). Nanoparticles carrying CpG ODN, or CpG ODNs that fold and aggregate to form nanoparticles, induce the production of higher amounts of interferon-α (IFNα) and -γ cytokines, which mediate the anti-viral response of plasmacytoid dendritic cells^20^,^21^,^22^,^23^,^24^. Interestingly, high interferon-α responses are similarly observed in the immune reaction to viruses. On the other hand, non-aggregating CpG ODNs are known to be poor interferon inducers, while being strong inducers of IL-6 production and expression of maturation markers on the cell surface^11^,^25^. This contrast results from the differing subcellular localization of nanoparticulate and soluble CpG ODNs in plasmacytoid dendritic cells and induction of distinct signaling pathways^26^.

All of these studies suggest that the morphology of a vehicle (virus or synthetic vaccine particle) has a strong effect in shaping the immune response raised by the biochemical signals (such as viral DNA or antigen) it carries. However, a systematic investigation of how the mammalian immune system responds to viral biochemical signals in the context of main viral shapes-spheres and rods-is not available in the literature. To answer this question, we compared the immune responses raised against CpG DNA delivered by zero- and one- dimensional nanostructures formed by the self-assembly of peptide amphiphile molecules and CpG ODNs (Fig. 1). These nanostructures resemble viruses in several ways: (i) their size and shape are comparable to viruses, where nanofibers resemble rod-like viruses and nanospheres are similar to spherical viruses, ii) they carry ODNs with motifs from viral DNA (CpG), which is known to activate TLR9 during viral infection^27^, and iii) they can be engineered to carry viral antigens through their peptide domains, which will allow repetitive organization of biochemical signals on their surface in a manner similar to viruses. As such, the present work concerns the design of virus-like nanostructures capable of potently eliciting anti-viral immune responses and the modulation of the immune responses against CpG DNA through changes in physical properties of the delivery system (Fig. 1).

## Results

### Design, synthesis and physical/chemical characterization of peptide nanostructures and their ODN complexes

**Figure 2:**
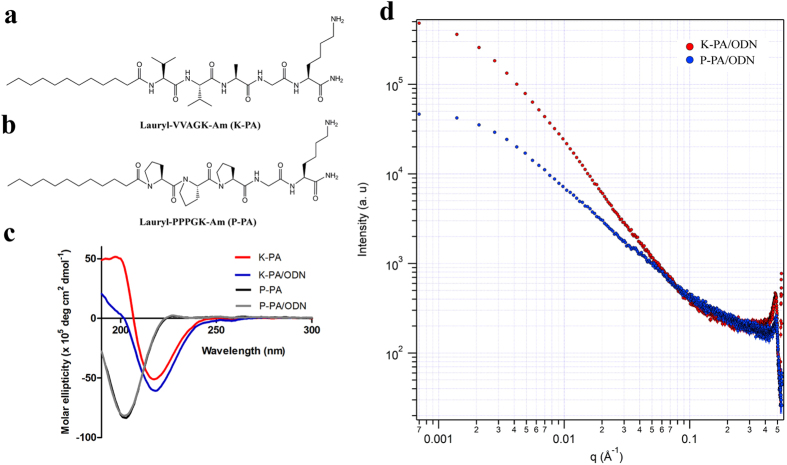
Structural characterizations of PA/ODN nanostructures. Chemical representations of (**a**) K-PA (Lauryl-VVAGK-Am) and (**b**) P-PA (Lauryl-PPPGK-Am). (**c**) CD spectra of PAs and PA/ODN complexes. (**d**) SAXS profiles of K-PA/ODN and P-PA/ODN complexes; y axis of the plot indicates scattering intensity, while x axis indicates scattering vectors.

We combined positively charged peptide amphiphile molecules with oppositely charged immunostimulatory oligodeoxynucleotides (CpG ODNs) to obtain one-dimensional (nanofibers) and zero-dimensional (nanospheres) self-assembled virus-like nanostructures. For this purpose, we synthesized two peptide amphiphile (PA) molecules with changing backbone motifs (K-PA and P-PA; Figs 2a,b and Fig. S1, S2) to direct self-assembled nanostructures into different morphologies. Both PA molecules include a lauryl group to drive self-assembly through hydrophobic collapse in aqueous solutions, a glycine residue as spacer and a lysine residue to confer them positive charge at physiological pH. The K-PA contains a Val-Val-Ala peptide sequence and showed characteristics of β-sheet organization (negative at 217 nm, positive at 197 nm) in circular dichroism (CD) spectra, while P-PA has a Pro-Pro-Pro peptide sequence and exhibits a PPII helix secondary structure (strong negative band at 203 nm and a weak positive band at 227 nm), similar to polyproline structures (Fig. 2c)^28^,^29^,^30^. Peptide/ODN complexes also preserved secondary structures of K-PA and P-PA and showed β-sheet and PPII helix characteristics in CD spectra, respectively (Fig. 2c).

Structural analysis of the peptide/ODN complexes were studied by using small-angle X-ray scattering (SAXS), which provides information on the shape and size of nanostructures in aqueous environment (Fig. 2d). The low q regions of the SAXS data of K-PA/ODN and P-PA/ODN complexes were best fitted into an elliptical cylinder model^31^ with a major radius of 8.6 ± 0.4 nm and oblate core shell sphere model^32^,^33^ with a major radius of 7.2 ± 0.3 nm, respectively. The results obtained from SAXS analyses showed that K-PA/ODN complexes self-assembled into one-dimensional, high-aspect-ratio elliptical cylindrical nanofibers, while P-PA/ODN complexes formed zero-dimensional oblate spherical nanostructures (Fig. S3a,b; Tables S1,S2)^34^. In addition, PDDF histograms of K-PA/ODN and P-PA/ODN complexes showed characteristic patterns of cylindrical and spherical nanostructures (Fig. S3c,d). For control experiments, K-PA and P-PA solutions at concentrations identical to peptide/ODN complexes analyzed by SAXS. SAXS profiles of K-PA and P-PA nanostructures were also best fitted to an elliptical cylinder model with a major radius of 8.0 ± 0.3 nm and oblate core shell sphere model with a major radius of 5.1 ± 0.3 nm, respectively (Fig. S4; Tables S3,S4).

**Figure 3:**
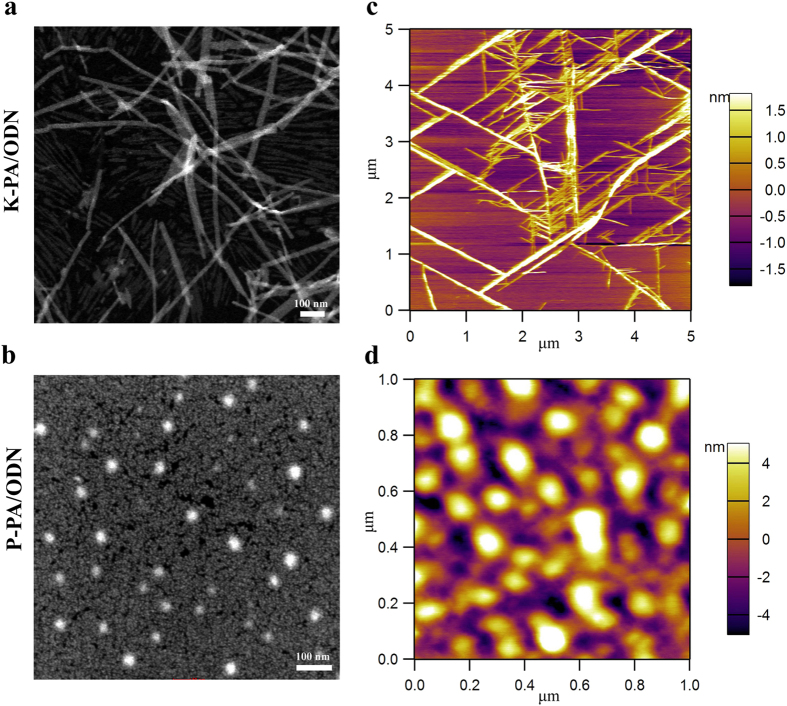
Imaging of PA/ODN nanostructures. TEM images of (**a**) K-PA/ODN and (**b**) P-PA/ODN self-assembled nanostructures. AFM images of (**c**) K-PA/ODN and (**d**) P-PA/ODN self-assembled nanostructures in aqueous environment.

STEM and AFM images showed that K-PA/ODN and P-PA/ODN complexes exhibited cylindrical and spherical morphologies in agreement with SAXS measurements (Fig. 3, Figs S5,S6,S8). In addition, the phosphorus signal obtained by EDX (energy-dispersive X-ray) spectroscopic analysis on spherical nanostructures and cylindrical fibers supported the interaction of ODNs with peptide molecules, and the formation of peptide/ODN complexes (Figs S5,S6). TEM images of the K-PA and P-PA nanostructures at the same concentrations with peptide/ODN complexes also showed cylindrical and spherical morphologies, as observed similar in SAXS measurements (Fig. S7). AFM imaging of the peptide/ODN complexes were performed to analyze the self-assembled systems in aqueous environment to eliminate the effects of drying on the sample imaging. The K-PA/ODN and P-PA/ODN complexes showed cylindrical and spherical morphologies, respectively, complementary to SAXS measurements and STEM imaging (Fig. 3c,d). The K-PA/ODN self-assembled into cylindrical bundles and larger aggregates of P-PA/ODN spherical complexes were observed in the aqueous environment. The aggregation and bundle formation on the AFM images in aqueous environment is related to the dynamic nature of peptide/ODN complexes and the self-assembly tendency of the peptides. In addition, AFM imaging of dried peptide/ODN complexes on the surfaces showed similar cylindrical and spherical morphologies of K-PA/ODN and P-PA/ODN complexes compared to aqueous environment at the same conditions (Fig. S8).

To distinguish the effect of nanostructure morphology on the immune response raised against CpG motifs from the immune response to the bare CpG motif itself, we prepared all groups with equal amounts of CpG ODNs (nanofiber, nanosphere and CpG ODN alone). Peptide concentrations were adjusted to ensure that all ODNs in solution bind to nanostructures. Since the negatively charged ODN interacts with positively charged lysine residues on peptide molecules, the number of CpG ODNs bound to nanostructures is directly related with the ODN to peptide ratio. As a result of PAGE analysis, we found that 1:70 and 1:2500 ratios were critical for ODN/K-PA and ODN/P-PA complexes, respectively (Fig. S9a,b). Zeta potential measurements were also consistent with PAGE results and indicated that similar ratios of ODN/peptide are critical for binding of all ODNs to nanostructures (Fig. S9c). The ODN solution had a zeta potential of −33 mV in the absence of the peptide molecules, due to highly negative charge of the ODN molecule (Fig. S9c). For the ODN/K-PA solution, we observed a slightly positive potential (+6 mV) at a 1:100 ratio, indicating the total neutralization of ODN with PA molecules. For ODN/P-PA, a ratio of 1:2500 was critical (+21 mV), similar to the ratio found in the PAGE experiment (Fig. S9c)^35^,^36^. Indeed, the zeta potential of the P-PA was several folds lower than an equimolar concentration of K-PA (Fig. S10). This suggests that at least some of the lysine molecules in P-PA are concealed in aggregates/nanospheres formed by P-PA. In the light of these results, we prepared ODN/K-PA and ODN/P-PA complexes at ratios of 1:100 ratio and 1:2500, respectively.

### Nanostructures protect ODNs from enzymatic degradation

**Figure 4:**
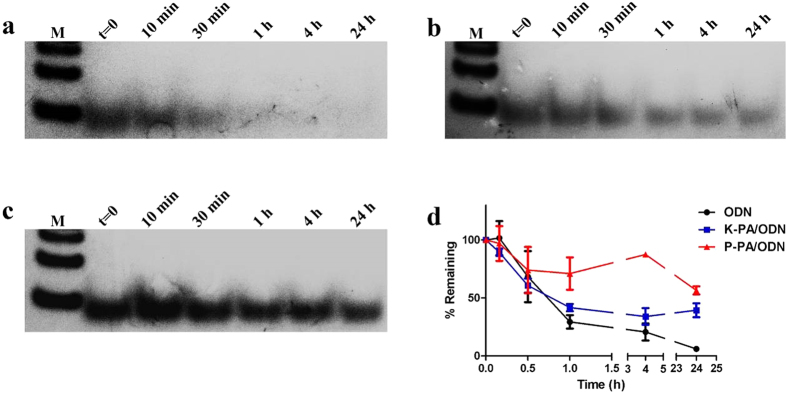
ODNs resist to enzymatic degradation better when bound to nanostructures. (**a**) ODN alone, (**b**) K-PA/ODN and (**c**) P-PA/ODN were treated with DNAse I for different time periods and, subjected to PAGE. Lane 1 is Marker, Lane 2 is non-treated ODN, Lane3-Lane7 = 10 min, 30 min, 1 h, 4 h and 24 h treatment with DNAse. (**d**) Time-dependent degradation of ODN in different formulations, plotted according to calculated band intensities.

Enzymatic degradation and consequent short plasma half-life are significant problems for the practical applications of CpG ODNs. We performed a DNAse assay to understand whether self-assembled PA nanostructures could enhance the stability of ODNs against enzymatic degradation. ODN alone, K-PA/ODN and P-PA/ODN groups were treated with DNAse I for different time periods. SDS detergent was used to remove ODNs bound to nanostructures after digestion. All samples were run on polyacrylamide gel for the visualizing remaining ODNs after DNAse treatment. As it is shown in Fig. 4a, ODN-alone was rapidly degraded and yielded no visible bands in gel after 30 min of DNAse treatment. Calculation of band intensities also revealed that ODN was almost completely degraded after 24 h of treatment – 6% remained according to the average of three samples (Fig. 4d). However, ODNs in K-PA/ODN or P-PA/ODN complexes were clearly observable even after 24 h of DNAse treatment (Fig. 4b,c). The P-PA/ODN system protected ODNs better than K-PA/ODN: 56% of ODNs remained after 24 h with P-PA/ODN, while 39% of ODNs remained with K-PA/ODN (Fig. 4d). These results indicated that nanostructures protect ODNs from degradation and that, nanospheres were more potent than nanofibers for this purpose.

### The shape of the CpG carrier is important for cytokine and co-stimulatory molecule expression profiles induced by CpG motifs

**Figure 5:**
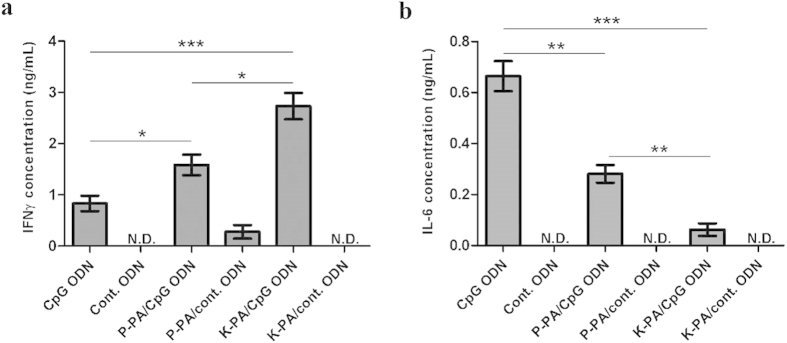
Nanostructures shift the CpG-induced cytokine secretion profile of splenocytes. Mouse splenocytes were treated with indicated formulations, and cytokine concentrations in culture media were detected with ELISA: (**a**) IFNγ, (**b**) IL-6. ODN concentration in all groups is 0.1 μg/mL. (*p < 0.05, **p < 0.01, ***p < 0.001 according to Student’s t-test) (N.D. is for “not detected”).

Spleen carries a wide variety of immune cells and can give insights into how various immune cells types would react to virus-like nanostructures. To evaluate the reaction of spleen cells to the virus-like shapes of ODN delivery agents, we treated cultured splenocytes with various doses of ODNs prepared as K-PA/ODN, P-PA/ODN and ODN alone (with same amount of CpG ODN in all groups to ensure accurate comparison). To exclude any CpG-free effect, we also treated cells under similar experimental groups with control ODN instead of CpG ODN (with reversed CpG motif, see Methods section). Cytokine production profiles of mouse spleen cells indicated that the direction of the immune response to CpG ODNs is modulated by the shape of nanostructured carrier systems. According to cytokine profiles, nanofibers (K-PA/ODN) triggered a highly Th1-biased immune response, as indicated by the elevated IFNγ and TNFα levels, which are significantly stronger than CpG ODN alone (300% and 50% increase, respectively) and nanospherical ODN complexes (P-PA/ODN) (100% and 50% increase, respectively) (Fig. 5 and Fig. S11). On the other hand, P-PA/ODN induced only a 50% higher IFNγ, which is a critical mediator of anti-viral response and is extensively involved in anti-tumor response, than CpG ODN alone (Fig. 5). When IL-6 expression, which is known to promote Th2 immune response, was analyzed, K-PA/ODN was found to be significantly weaker than CpG ODN alone and P-PA/ODN. P-PA/ODN also induced diminished IL-6 production when compared to CpG ODN alone (Fig. 5b and Fig. S11b). On the other hand, IL-12 induction with K-PA/ODN or P-PA/ODN was not significantly different from ODN alone, indicating that higher IFNγ induction with K-PA/ODN and P-PA/ODN was not IL-12 dependent (Fig. S11d). ODNs lacking CpG motifs were not effective in general, even in the presence of nanostructures, which indicates that the altered immune response provided by nanostructures was still CpG-dependent (Fig. 5). At concentrations lower than 0.1 μg/mL, P-PA/ODN preserved its potency of IFNγ production better than K-PA/ODN and ODN alone (Fig. S11a).

**Figure 6:**
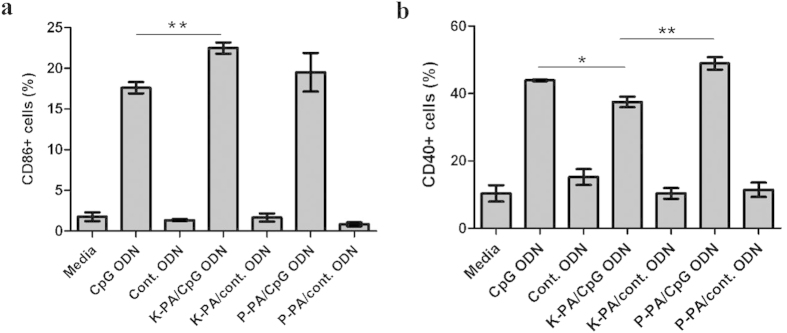
Effect of nanostructures on the CpG-induced surface expression of co-stimulatory molecules. Mouse splenocytes were treated with indicated formulations for 24 h and percentage of cells expressing (**a**) CD86 or (**b**) CD40 in total population were detected by flow cytometry. (*p < 0.05, **p < 0.01 according to Student’s t-test).

The upregulation of co-stimulatory molecules on the surfaces of immune cells is another mechanism of immune activation induced by pathogenic signals such as CpG DNA. To understand the effect of nanostructures on this process, we cultured splenocytes in the presence of K-PA/ODN, P-PA/ODN and ODN alone in a similar fashion to the cytokine assay and checked the expression levels of the cell surface markers; CD86 and CD40 using flow cytometry. We observed that the amount of CD86 expressing cells in splenocytes is upregulated better by K-PA/ODN compared to ODN alone, and that, the effect is strictly CpG-signal specific, as nanofibers with control ODNs proved to be non-effective (Fig. 6a). Treatment with P-PA/ODN induced CD86 expression in a higher percentage of cells compared to ODN alone, while the difference was not statistically significant (Fig. 6a). Regarding CD40 expression profiles, while all CpG-containing formulations induced CD40 expression (30–40%) in splenocyte population better than control ODN (~10%), P-PA/ODN showed the highest signal (48%). In addition, ODN alone induced a higher (44%) number of cells to express CD40 than K-PA/ODN (37%). Overall, nanostructures synergize with the ODN in the induction of co-stimulatory molecule expression, and nanostructure morphology makes a substantial difference where nanofibers induce CD86 expression more than nanospheres, which induce CD40 expression better than nanofibers (Fig. 6b).

### PA/ODN complex stability does not explain the differential immune responses induced by nanofibers and nanospheres

One alternative explanation for the different immune profiles induced by spherical and fibrous nanostructures is their differential stability in cell culture media. To determine whether this factor was responsible for modulating the immune response, fluorescent-tagged ODNs (FITC-ODN) were incubated in cell culture media for 48 h either alone or as K-PA/ODN and P-PA/ODN complexes and changes in fluorescence were recorded for this time period. Both K-PA/ODN and P-PA/ODN showed decreased fluorescence when compared to bare ODNs, indicating partial quenching due to the aggregation of ODNs on fibers or spheres (Fig. S12). However, RFU (relative fluorescence unit) values for both K-PA/ODN and P-PA/ODN did not change considerably during the course of the experiment, excluding differential stability as a potential cause of the differences observed.

### Nanofibrous carriers accelerate the internalization of ODNs into specific immune cells

**Figure 7:**
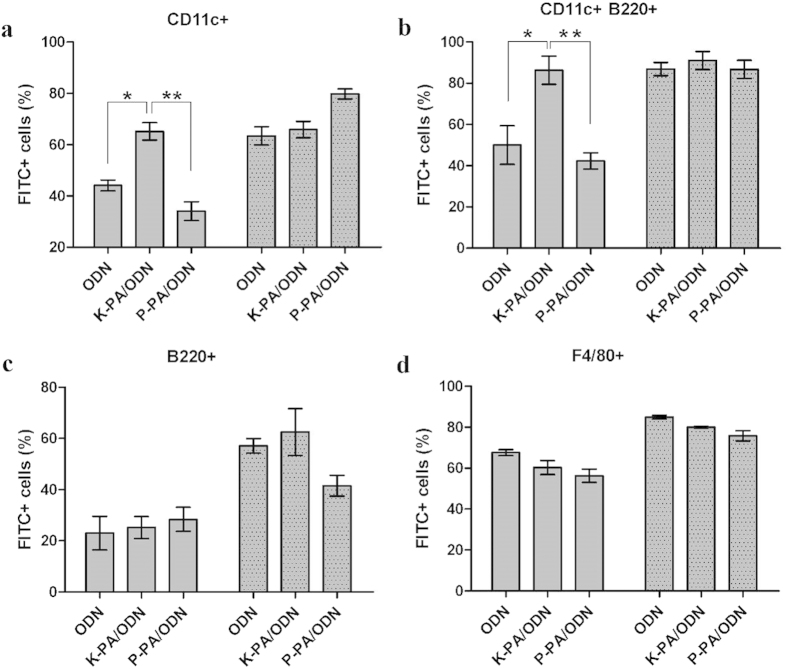
Uptake profiles of FITC-ODN alone or bound with nanostructures into TLR9+ cell subsets in mouse splenocytes. Percentage of FITC (ODN)+ cells (**a**) in CD11c+ (dendritic cells), (**b**) CD11c+B220+ (plasmacytoid dendritic cells), (**c**) B220+(B cells) and (**d**) F4/80+ (macrophages) populations. Gray and dotted gray bars indicate 2 h and 12 h culture with ODN formulations, respectively. (*p < 0.05, **p < 0.01 according to Student’s t-test).

To analyze the uptake of ODNs into different immune cells, we prepared nanofibrous and nanospherical complexes by using FITC-conjugated CpG ODNs. Mouse splenocytes were cultured with ODN-FITC alone, K-PA/ODN-FITC and P-PA/ODN-FITC for two different durations (2 and 12 h). In all types of cells investigated, the uptake of ODN, alone or bound to nanostructures, increased as a function of time, except for K-PA/ODN uptake into dendritic (CD11c+) and plasmacytoid dendritic (CD11c+B220+) cells (Fig. 7). These results indicate that 2 h is not sufficient for maximal uptake of ODN alone into relevant immune cells. However, K-PA/ODN achieved maximal uptake into dendritic cells (DC) and plasmacytoid dendritic cells (pDC) at 2 h (Fig. 7a,b). At both 2 h and 12 h, K-PA/ODN positive cells in DC and pDC populations were about 65% and 85–90%, respectively. ODN alone or P-PA/ODN achieved this level of uptake at 12 h. At 2 h, ODN was internalized by 44% and 50% of DCs and pDCs, while P-PA/ODN was internalized by 34% and 42% of these cells, respectively (Fig. 7a,b). Previously, removal of CpG ODNs from cellular culture media before 8 h of culture was reported to reduce its immune activating potential, which suggests that time is an important factor for the entry of CpG ODNs into TLR9-positive endosomes^37^.

All of the groups were internalized similarly to B-cells (B220+) and macrophages (F4/80+) (Fig. 7c,d). The uptakes of ODN and K-PA/ODN (57% and 63%, respectively) to B-cells were higher at 12 h than P-PA/ODN (42%), while only the difference between ODN alone and P-PA/ODN was statistically significant (Fig. 7c).

### Internalization mechanism of ODN complexes

To understand the internalization mechanisms involved in CpG ODN, K-PA/CpG ODN, and P-PA/CpG ODN uptake, we prepared these complexes by using FITC-conjugated CpG ODNs. RAW 264.7 macrophages were cultured for 2 h with each endocytosis inhibitor (amiloride, nocodazole, chloropromazine, nystatin, and cytochalasin D), then the medium was removed and treated with FITC-CpG ODN, K-PA/ FITC-CpG ODN, and P-PA/FITC-CpG ODN containing medium for 2 h. Table S6 shows the pathway affected by each chemical inhibitor and the protein target that is altered after its binding. Figure S14 shows effects of each inhibitor on CpG ODN and bound to nanostructures internalization by RAW 264.7 cells. Cytochalasin D treatment induced significant reduction in uptake for all forms of CpG ODN, thus pointing to macropinocytosis mechanism takes the role for the internalization of all kinds of CpG complexes. On the other hand, amiloride, nocodazole and chlorpromazine seem to increase the uptake of FITC-CpG ODN, K-PA/ FITC-CpG ODN, and P-PA/FITC-CpG ODN through other mechanisms probably due to cyoskeletal alterations which may result in higher internalization. Moreover, nystatin did not alter uptake profile of CpG ODN and its nanostructure forms. Finally, similar to uptake study with splenocytes (Fig. 7a, b), nanofibers are superior to nanospheres and CpG ODN in terms of fast internalization.

### CpG ODNs synergize with nanofibers to induce antigen-specific IgG

**Figure 8:**
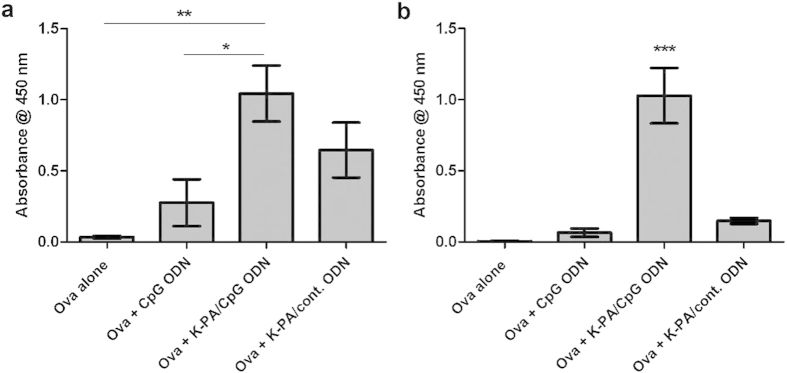
Antigen-specific IgG levels for each immunized group. (**a**) Day 13 IgG levels (1/100 diluted sera) after primary injection; *p < 0.05, **p < 0.01. (**b**) Day 28 IgG levels (10^−5^ diluted sera) 13 days after secondary injection; ***p < 0.001 between Ova + K-PA/CpG ODN and each of other groups. One-way ANOVA with Tukey’s multiple comparison test was used for statistical evaluation.

Since nanofibrous ODNs showed remarkable potential for inducing the Th-1 immune response in *in vitro* experiments, their potency as a vaccine system was also investigated *in vivo*. Balb/c mice were immunized twice (day 0 and 15) with a model antigen (Ova) either alone or with adjuvant formulations. Blood was collected from animals at two time points (day 13 and 28) and Ova – specific IgG levels were analyzed from isolated sera. These data convincingly indicated that “Ova with K-PA/ODN” vaccine system is superior to “Ova with CpG ODN” system (Fig. 8 and Fig. S13). Even after the first immunization (day 13 samples), “Ova with K-PA/ODN” showed significantly higher IgG production than “Ova with CpG ODN” and “Ova only” systems (Fig. 8a and Fig. S13a). While “Ova only” induced barely above of undetectable amount of IgG at 100-fold dilution of sera, IgG signal in sera from “Ova with K-PA/ODN”-treated animals declined to similar level only at 10^4^ dilutions of sera (Fig. S13a). The difference between “Ova with K-PA/CpG ODN” and “Ova with K-PA/control ODN” did not reach statistical significance in day 13 samples (Fig. 8a). In serum samples obtained after booster immunization, the difference between the IgG signals of “Ova with K-PA/CpG ODN” and other groups increased. While other groups gave either undetectable or barely detectable IgG signals at 10^5^ dilution of serum, “Ova with K-PA/CpG ODN” induced a strong IgG signals (Fig. 8b). Overall, day 28 serum titration curves for IgG signals showed that “Ova with K-PA/CpG ODN” induced more than 10-fold IgG than “Ova with CpG ODN” or “Ova with K-PA/control ODN” and more than 100-fold IgG than “Ova only” systems (Fig. S13b). Since CpG ODN amounts are identical in both “Ova with CpG ODN” and “Ova with K-PA/CpG ODN”, it can be concluded that the nanofibrous presentation of CpG ODN elevates specific adaptive immune response against the co-delivered antigen. Moreover, the effect of CpG ODN and the nanofiber morphology is not additive but synergistic in “Ova with K-PA/CpG ODN”. As the effect of both stimuli presented in tandem, 10-fold higher IgG response than “Ova with CpG ODN” or “Ova with K-PA/control ODN” was observed.

## Discussion

Functional self-assembled architectures with varying morphologies can be tailored by using simple molecular building blocks with distinct characteristics^38^. The specific attributes of different amino acids guide the self-assembly of peptide molecules and determine their structural properties. Valine and alanine residues found in peptide molecules are known as “β-sheet formers”, and favor the formation of one-dimensional nanostructures, while proline residues are called “β-sheet breakers”, result in the self-assembly of zero-dimensional nanostructures^28^,^29^,^39^. In addition, the self-assembly of peptides into one or zero dimensional nanostructures can be promoted by mixing oppositely charged biomacromolecules. Non-covalent interactions such as hydrogen bonding, electrostatic and hydrophobic interactions between the peptides and biomacromolecules enable the formation of supramolecular assemblies^40^,^41^,^42^. Peptide amphiphile (PA) molecules were designed according to these principles, and their complexes with immunostimulatory ODNs were consequently able to self-assemble into nanospheres and nanofibers. In addition to designing new platforms for CpG ODN delivery, we also aimed to understand whether the shape of delivery system had any influence on the immune response. Both nanospheres and nanofibers showed potency in protecting ODNs from nuclease degradation, making them promising delivery platforms. Nanostructure binding possibly makes ODNs less accessible to enzymes, which might be the mechanism responsible for the protection.

Qualitative change in immune responses were observed when A-type and B-type CpG ODNs were delivered in the presence of peptide nanostructures. A-type ODNs, which have polyG sequences at both ends, form higher-order structures through the folding of their palindromic sequence and hydrogen-bonding of guanosines. These ODNs manifest themselves as stable nanoparticles of about 20–100 nm in size, which is remarkably similar to spherical viruses^21^. On the other hand, B-type ODNs (such as ODN1826 used in this study) do not have similar palindromic sequences and polyG sequences, and hence do not form any higher-order structures. A-type ODNs or nanoparticle-bound B-type ODNs were shown to induce several folds higher IFNγ and lower IL-6 production from immune cells than B-type ODNs^20^,^21^,^43^. Moreover, A-type ODNs drive the Th1 development of naive CD-4 T-cells to greater extent than B-type ODNs^44^. Elevated IFNγ production from PBMCs (peripheral blood mononuclear cells) by CpG ODNs with nanoparticulate structures (A-type ODN) was shown to be IFN-α dependent^44^. This change in immune profile was explained by the longer retention of nanoparticulate ODNs in the early endosomes of plasmacytoid dendritic cells compare to linear ODNs and inducing MyD88–IRF-7 signaling pathway^26^, while B-ODNs rapidly localize to lysosomes and induce the MyD88– NF-κB pathway. Our CpG ODN-containing spherical particles morphologically resemble nanoparticles used in these studies (both can be called as zero-dimensional) and similarly induced higher IFNγ and lower IL-6 responses than B-type CpG ODNs. Importantly, our findings also reveal that one-dimensional nanofibers synergize with CpG ODNs better than nanospheres in terms of IFNγ and TNFα activation, and inhibition of IL-6 production, which indicates that immune response is driven further to the Th1 direction. To the best of our knowledge, this is the first report showing tunable nature of immune response to pathogenic DNA motifs by changing shape of the carrier nanostructures.

Nanostructure shape also influences the CpG-induced expression of the surface molecules– CD86 and CD28. These molecules are expressed by antigen presenting cells (APC) upon sensing infection and play a vital role in the development of the adaptive immune response against foreign antigens. The interaction of CD86 with CD28 on T-cell surfaces is required for recognition of the foreign antigens and activation of T-cells during antigen presentation to T-cell receptors. CD40 binding to target cells induces B-cell and macrophage activation and the differentiation of B-cells. Surface molecule expression data therefore suggest that the shape of nanostructures carrying CpG motifs affects the nature of the adaptive immune response.

Recent studies have shown that the shape of particle significantly modifies the mechanism and extend of its cellular uptake. High-aspect-ratio PEG particles were internalized into HeLa cells four times faster than low-aspect-ratio particles and were rapidly translocated into nuclear membrane^45^. In another work, the shape of the particle surface at the point of initial contact with macrophages was shown to determine whether it would be phagocytosed^46^. Polystyrene particles with oblate ellipsoid shapes displayed higher internalization and phagocytosis into macrophages than prolate ellipsoid and spherical particles^47^, while prolate ellipsoid particles were better in cellular binding. Barua *et al.* showed that polystyrene nanorods coated with antibodies specific to cellular receptors exhibit higher cellular (HER2-expressing breast cancer cell line) binding, uptake and bioactivity than polystyrene nanospheres^48^. However, regarding non-specific uptake (*i.e.* no specific antibody on nanostructure to bind cellular receptors), rods were inferior to spheres. This might be due to the fact that rods have larger contact area with cellular surfaces, which cause higher surface adhesion through receptor-ligand interactions^48^. Considering these studies, we hypothesized that differential responsiveness of immune cells to K-PA/ODN, P-PA/ODN and ODN alone might be caused by differential uptake into immune cells. Overall, our results strongly suggest that nanofibrous structures accelerate the uptake of CpG ODNs into DCs and their subset – pDCs than their counterparts. The increased uptake of K-PA/ODN to pDCs might be an explanation for their capability to induce IFNγ. As mentioned above, IFNγ production can be induced by IFNα, which is released from pDCs upon stimulation with nanoparticulate CpG ODNs^44^. Hence, better uptake of nanofibrous ODNs to these cells might be related to their unexpectedly high Th1-biased immune response activating potential. Previously, the scavenger receptor CXCL16 of plasmacytoid dendritic cells was suggested to bind nanoparticle-forming D-ODN (analogue to A-ODN) molecules, facilitating their cellular uptake and modifying resulting immune responses, such as the IFNα response^49^. Similar receptors might be functioning for the recognition of the nanofiber structure and its preferential uptake into DCs and pDCs. It is feasible that the larger contact area of nanofibers allows them to spread across the cell surface and cross-links membrane receptors that are important for uptake, allowing their internalization to occur better than the spherical PA/ODN complex or ODN alone.

Better uptake into relevant cells and increased nuclease resistance are among the possible factors that provide an advantage to K-PA/CpG ODNs over bare CpG ODNs for inducing the immune response *in vivo*. However, the CpG signal is still vital for the function of complex, as the K-PA/control ODN induced an immune response significantly weaker than its CpG-bearing counterpart (Fig. 8b and Fig. S13b). CpG-mediated signaling and the concomitant increase in the expressions of cytokines and co-stimulatory surface markers possibly induce B-cell differentiation, maturation and antibody secretion. Ova encapsulation into nanofibers probably also contributes to the immune response, since “Ova with K-PA/control ODN” caused similar IgG signal with “Ova with CpG ODN” (Fig. 8b and Fig. S13b). To assess the potential toxicity of K-PA/ODN complexes, we measured the viability of splenocytes treated with these nanostructures. Increasing CpG ODN dose positively contributed to the viability of splenocytes in both ODN alone and K-PA/ODN groups (Fig. S16). This suggests that CpG ODNs exhibit a proliferative effect on immune cells, mainly B-cells, since control ODN groups did not show such statistically significant changes in any concentration of ODN (Fig. S16). We also observed similar increase in the viability of cells treated with K-PA/CpG ODN group, while no significant difference was observed between the viabilities of cells treated K-PA/control ODN and non-treated cells (Fig. S16). These results clearly exclude the toxic effect of K-PA/ODN as a component in the resulting immune response. On the other hand, P-PA/ODN complexes significantly reduced the viability of splenocytes at the concentrations used for *in vivo* assays, thus we decided to exclude this group from the *in vivo* experiments (Fig. S16).

There are many reasons for the misguiding experimental results built in preparation of the nanomaterials. These concerns should be addressed with the appropriate controls to exclude their effect. These sources are mentioned as endotoxins or other contaminants, solvents and dispersion agents and their dosage, agglomeration of nanoparticles at higher concentrations, cross-reaction of nanoparticles with analytes in the test system^50^. Especially, endotoxin or other microbial contamination may lead to inflammatory response interfering with our immune response data. We used nanostructures with immunologically neutral (GpC) ODN as a control, which includes same peptide molecules for nanostructure formation, same solvent and other possible interfering sources as test system. Both nanofiber and nanosphere formation was similar between CpG or control ODN molecules hence agglomeration effects should be similar between the groups. Taking these into consideration, we can suggest that the difference between nanofibers, nanospheres and soluble bare ODN groups is due to the interaction between specific nanostructure morphology and ODN.

Here, we demonstrated that immune response against viral/bacterial DNA patterns depends strongly on the shape of the carrier nanostructure. As a delivery system, nanofibers were more effective than nanospheres in driving the CpG-induced immune response towards the Th1 phenotype, which is specialized for defending against intracellular pathogens. Nanofibrous ODN complexes also showed enhanced activity in *in vivo* experiments compared to ODN-antigen and antigen alone treatments. Complexes with soluble model antigens induced the production of specific immunoglobulins better than the administration of antigen with CpG ODNs. The versatility of the peptide nanosystems can enable the engineering of nanostructures to carry viral, bacterial or tumoral antigenic peptides. Delivering antigenic peptide and CpG ODN adjuvant in close proximity would also increase the efficiency of these nanofibers for inducing robust antigen-specific humoral and cellular immune responses.

## Methods

### Materials

9-Fluorenylmethoxycarbonyl (Fmoc) and tert-butoxycarbonyl (Boc) protected amino acids, [4-[α-(20,40-dimethoxyphenyl) Fmoc-aminomethyl] phenoxy] acetamidonorleucyl-MBHA resin (Rink amide MBHA resin), and 2-(1Hbenzotriazol-1-yl)-1,1,3,3-tetramethyluronium hexafluorophosphate (HBTU) were purchased from NovaBiochem and ABCR. Other chemicals for peptide synthesis were purchased from Fisher, Merck, AlfaAesar, or Aldrich. All chemicals were used as provided. CpG and control ODNs were purchased from Invivogen. Paired antibodies and recombinant proteins of IFNγ and IL-12 were obtained from R&D systems, that of IL-6 from eBioscience and TNFα from Life Technologies. All cell culture and ELISA reagents were purchased from Life Technologies, except non-essential amino acid solution (Sigma Aldrich). Reagents for polyacrylamide gel electrophoresis were obtained from Sigma Aldrich. Fluorescently labelled antibodies were obtained from BD Pharmingen (B220 and CD11c) and eBioscience (F4/80, CD40, CD86).

### Peptide synthesis

Lauryl-VVAGK-Am (K-PA) and Lauryl-PPPGK-Am (P-PA) were synthesized on Rink Amide MBHA resin. Amino acid couplings were performed with 2 equivalents (equiv) of Fmoc-protected amino acid, 1.95 equiv of HBTU and 3 equiv of N,N-diisopropylethylamine (DIEA) for 2 h. To remove the Fmoc group, 20% (v/v) piperidine/dimethylformamide solution (DMF) was added and the resin incubated for 20 min. To block the remaining free amine groups after amino acid coupling, 10% (v/v) acetic anhydride solution in DMF was used (30 min). After each step, the resin was washed using DMF, dichloromethane (DCM) and DMF. A trifluoroacetic acid (TFA)/triisopropyl silane (TIS)/H_2_O/DCM mixture (5:2.5:2.5:90 ratio) was used to cleave the peptide from the resins.

### Preparation of virus-like nanostructures

Virus-like nanostructures were prepared through the self-assembly of peptide molecules in the presence of oligonucleotides. To form one-dimensional nanofibrous and zero-dimensional nanosphere structures, positively charged K-PA and P-PA (Fig. 2a,b) molecules were mixed with CpG (ODN1826) or control ODNs, respectively. Two CpG motifs in ODN1826 were reverted in the control ODN sequence: ODN1826: 5′-tccatga**cg**ttcctga**cg**tt-3′; ODN1826 control: 5′- tccatga**gc**ttcctga**gc**tt -3′. The exact molar ratio for ensuring that all ODNs in solution interact with nanostructures was determined to be 100:1 for K-PA/ODN and 2500:1 for P-PA/ODN (Fig. S9). Nanostructures were prepared with these ratios for all experiments and called nanofibrous ODN (K-PA/ODN) and nanospherical ODN (P-PA/ODN) throughout the manuscript. In all experiments, at least three independent nanofiber and nanosphere formulations were prepared and tested.

### Small Angle X-ray Scattering (SAXS) analysis of self-assembled nanostructures

The PA/ODN complexes were prepared by mixing an ODN1826 solution (15 μg/mL) with identical volume of 0.375% (w/v) P-PA solution (2500:1 ratio) or 0.015% (w/v) K-PA solution (100:1 ratio). The final ODN concentration in each PA/ODN complex was equal. For control experiments, 0.008% (w/v) K-PA and 0.188% (w/v) P-PA solutions were prepared. Each PA/ODN, K-PA and P-PA solution was loaded into a quartz capillary cell for SAXS measurement. SAXS measurements were performed with a Kratky compact HECUS (Hecus X-ray systems, Graz, Austria) system equipped with a linear collimation system and X-ray tube Cu target (λ = 1.54 Å). The generator was operated at a power of 2 kW (50 kV and 40 mA). Simultaneous measurements of SAXS and WAXS range are possible in the system with a linear-position sensitive detector used with 1024 channel resolution. Inter-channel and sample-detector distances were 54 μm and 31.5 cm, respectively. Scattering curves were monitored in q ranges of 0.004–0.55 Å^−1^ for SAXS and 1.03–2.15 Å^−1^ for WAXS. All peptide/ODN complexes, K-PA and P-PA solutions were measured for 900 s at room temperature (23 °C).

### Transmission Electron Microscopy (TEM) imaging

Nanostructures were imaged by TEM as follows (Fig. 3a,b and Figs S5–S7). 30 μL of PA/ODN complexes was prepared on parafilm by mixing 15 μL of 15 μg/mL ODN1826 with 15 μL of either 0.375% (w/v) P-PA (2500:1 ratio) or 0.015% (w/v) K-PA (100:1 ratio). For PA-only samples, these concentrations of PAs were mixed with distilled water instead of ODN solution. TEM grids were inverted onto these solutions. Grids were removed after 5 min and the remaining solution on grid was absorbed by a lint-free paper. Staining was performed with 2% (w/v) uranyl acetate solution (Ted Pella, Inc) for 1 min. Grids were then immersed into ddH_2_O once and dried overnight at room temperature. TEM imaging was performed on the next day by a FEI, Tecnai G2 F30 instrument. All images were taken in STEM mode with a high angle annular dark field (HAADF) detector.

### Atomic Force Microscopy (AFM) imaging

PA/ODN complexes for AFM imaging were prepared in liquid or dried conditions. ODN1826 solution at 15 μg/mL concentration was mixed with an identical volume of 0.375% (w/v) P-PA solution (2500:1 ratio) or 0.015% (w/v) K-PA solution (100:1 ratio). The final ODN concentration in each PA/ODN complexes was equal. For K-PA/ODN complexes, the prepared solution was diluted 50 times and dropped onto the cleaned mica surface and imaged directly in aqueous environment (Fig. 3c) or dried overnight and, imaged on the mica surface (Fig. S8a). SiN soft contact tip was used for contact mode imaging of K-PA/ODN complexes. For P-PA/ODN complexes, the solution was diluted 100 times, dropped onto the cleaned glass surface and imaged directly in aqueous environment (Fig. 3d) or dried overnight and imaged on the glass surface (Fig. S8b). Si tip (150 kHz, k = 5 N/m) was used for soft-tapping mode imaging of P-PA/ODN complexes. MFP3D Asylum microscope was used for imaging.

### Circular Dichroism (CD) Spectroscopy

CD spectroscopy was performed with a JASCO J815 CD spectrometer at room temperature. 0.2 mM solutions of both K-PA and P-PA and their mixtures with ODN1826 (100:1 and 2500:1, respectively) were measured from 300 to 190 nm. Data pitch was 1 nm, scanning speed was 100 nm/min, and all measurements were performed with three accumulations. DIT was selected as 4 s, bandwidth as 1 nm, and the sensitivity was as standard. Molar ellipticity was calculated using the equation: [θ] = 100 × θ/(C × l), where C is the molar concentration, and l is the cell path length in centimeters. [θ] = θ/(C × l) = deg/(mol/1000 cm^3^) × 0.1 cm = 100 deg cm^2^ dmol^−1^.

### Polyacrylamide Gel Electrophoresis (PAGE)

PAGE was performed to identify the critical ODN/PA ratio required to conjugate all ODNs in solution to PA nanostructures. 20 μg/mL ODN1826 solution (15 μL) was mixed with varying concentrations of PA solutions (15 μL) to prepare different ODN/PA ratios (from 1:10 to 1:2500). These solutions were mixed with Orange DNA loading dye (Fermentas) and loaded onto 20% polyacrylamide gels. 10 μL of 10 bp DNA ladder (O’range ruler^TM^, Fermentas) was used as marker. Gels were run at 75 V for 1 h and subsequently at 50 V for 2.5 h (in 1x TAE). Stains-all dye working solution (0.005%, w/v) was prepared freshly from stock solution (0.1% w/v) as recommended by manufacturer (Sigma Aldrich). Gels were incubated in Stains-all overnight (dark conditions and room temperature). On the next day, the destaining of gels was performed under sunlight and images were taken by a Nikon camera.

### Zeta Potential Measurements

Zeta potential measurements were performed to find critical ratio of ODN/PA at which, all ODNs in solution were neutralized by (and bound with) PAs. 400 μL of 5 μg/mL ODN1826 solution was mixed with varying concentrations of PA solutions (400 μL) to prepare different ODN/PA ratios (from 1:10 to 1:2500). Zeta potentials of these solutions were measured with a Nano-ZS Zetasizer (Malvern). Measured mobility was converted to zeta potential using the Smoluchowski equation. All measurements were performed in triplicate – by using three independently generated formulations.

### Animals

All experimental procedures involving animals were approved by the Animal Ethics Committee of Ankara Diskapi Yildirim Beyazit Training and Research Hospital (Protocol # 2013/25). Primary spleen cells were obtained from adult BALB/c (12–16 weeks old) mice, which were maintained under controlled conditions and fed *ad libitum*.

### Splenocyte culture and stimulation experiment

Spleens were removed aseptically and grinded between a petri plate surface and the plunger end of a syringe in culture media (2% FBS in RPMI-1640) in order to dissociate single cells from bulk tissue. Single cell suspension was collected carefully to exclude tissue debris. Cell suspension was centrifuged at 800 g for 10 min. Supernatant was discarded and cell pellet was resuspended in culture medium (this step was performed twice). Cells were adjusted to 2 × 10^6^ cells/mL cell density and cultured in 96-well plates as 200 μL/well (4 × 10^5^ cells/well). The medium used for the splenocytes culture was composed of RPMI-1640 with 5% FBS (Pen/Strep, L-Glu, non-essential amino acids and HEPES (20 mM) were also added). Cell stimulation was performed immediately after distributing cells to wells. K-PA/ODN and P-PA/ODN were prepared as described above by using different doses of ODN1826 or control ODN. Nanostructure and ODN-only solutions were further diluted with media and final concentration of ODN in cell suspension was in a range of 1 μg/mL to 0.01 μg/mL. For cytokine analysis, cells were cultured at 37 °C and 5% CO_2_ for 48 h and supernatants were collected at the end of the experiment. For the analysis of surface markers (co-stimulatory molecules), cells were treated with same formulations (under an ODN dose of 0.3 μg/mL) for 24 h. Cells were collected at the end of experiment for further staining and analysis by flow cytometry. All experiments outlined were performed in triplicate; representative results of three independent experiments are shown.

### ELISA

Cytokine concentrations in supernatants collected from cultures at the end of the splenocyte stimulation experiment were measured by ELISA. MaxiSorp^TM^ plates (Thermo Scientific, NUNC) were coated with IL-6, IL-12 or IFN-γ primary antibodies (overnight incubation at 4 °C). On the next day, plates were blocked with 0.5% BSA (2 h), incubated with supernatants of cell culture experiment or standard recombinant proteins (2 h), biotin-labeled secondary antibody (2 h) and HRP (horse radish peroxidase)-conjugated streptavidin (1 h), consecutively, at room temperature. Plates were washed 5 times with washing buffer and dried by tapping between each consecutive steps (except the first two steps, in which washing was performed once per step). TMB (3,3′,5,5′-Tetramethylbenzidine) substrate was added at the last step and reaction was stopped after 15–20 min by 1.8 N H_2_SO_4._ Color formation was measured by microplate reader (Spectramax M5, Molecular Devices) as absorbance at 450 nm wavelength. This value was subtracted from a reference value (650 nm) to obtain absorbance values attributable solely to dye color. All treatments were performed with at least three replicates and shown as mean +/− standard deviation.

### Assessment of the effect of nanostructures on cell viability

MTT-based *in vitro* toxicology assay kit (Sigma Aldrich) was used for the assessment of cell viability. Splenocytes were treated with ODNs or their peptide complexes (nanofibers and nanospheres) under conditions described in the stimulation assay for 36 h. After 5 min of centrifugation, culture media were discarded and cells were incubated in medium with MTT reagent (10%) for 4 h. Crystals formed in wells were dissolved with solubilization reagent and absorbance values were measured with microplate reader (Spectramax M5, Molecular Devices).

### Evaluation of the stability of PA/ODN complexes

ODN-FITC, K-PA/ODN-FITC and P-PA/ODN-FITC were prepared (n = 2) and incubated in cell culture media under conditions described in the stimulation assay (without cells in this case). Samples were taken at certain time points and fluorescence (ex: 495, em: scanned between 450–650 nm) was measured using a NanoDrop3300 fluorospectrometer.

### Internalization of ODNs into immune cells

Internalization of ODNs into various immune cells expressing TLR9 in total splenocytes was analyzed by flow cytometry. For this purpose, FITC-conjugated ODN was used for preparing K-PA/ODN and P-PA/ODN. Freshly prepared mouse splenocytes were cultured in 96-well plates (4 × 10^5^ cells/well). Cells were treated with K-PA/ODN, P-PA/ODN or ODN alone for 2 h or 12 h before flow cytometry experiment. Cells were collected into 1.5 mL Eppendorf tubes by pipetting, and precipitated by centrifugation. Supernatants were discarded, cells were washed with 1x PBS and a cell pellet was obtained again by centrifugation for further staining and analysis by flow cytometry.

### Internalization mechanism of PA/ODN complexes

Chemical inhibitors for various internalization pathways were used to understand the internalization mechanism of PA/ODN nanostructures. Optimal inhibitor concentrations that give maximal inhibition of relevant pathway but yet non-toxic to cells were determined. Raw 264.7 cells were seeded in 6 well plates (1 × 10^6^ cells/well) with 10% FBS DMEM. After 8 h from seeding, chemical inhibitors were administered in fresh medium at final concentrations of 1 mM amiloride, 25 μg/mL nystatin, 5 μg/mL nocodazole, 10 μM cytochalasin D, and 2 μg/mL chloropromazine. After 2 h of incubation, cells were rinsed gently with fresh medium to remove chemical inhibitors. FITC-conjugated CpG was used for preparing K-PA/ODN and P-PA/ODN. Cells were treated with K-PA/ODN, P-PA/ODN and bare CpG ODN for 2 h before flow cytometry experiment. After 2 h incubation with nanostructures, cells were rinsed with 1x PBS three times to remove non-internalized nanostructures, and collected into Eppendorf tubes with scraper. Then, they were precipitated with centrifugation at 2500 rpm for 5 min, cells were dissolved in 1x PBS. After scraping, cells were kept on ice until flow cytometry analysis. Internalization in macrophage cells were analyzed with BD Accuri C6 flow cytometer (BD Biosciences). Cells were gated by SSC (side scatter channel) and FSC (forward scatter channel) using non-treated control. Fluorescence intensity of cells was measured with green channel.

### Staining of surface markers and flow cytometry

For uptake study, cells were stained with anti-B220-PE and anti-CD11c-APC or anti-F480-PE. For analysis of the expression of co-stimulatory molecules, cells were stained with CD40 and CD86 antibodies. Cells were washed with 1X PBS and centrifuged twice and resuspended in 1x PBS. Flow cytometry was performed with BD FACSAria^TM^ III equipment with BD FACSDiva^TM^ software. The number of events was at least 10,000 for all samples. The experiment was performed in triplicate and representative results of two independent experiments are shown.

### DNAse assay

A DNAse assay was performed to understand whether nanostructure binding protects ODN from enzymatic degradation (Fig. 7). Briefly, K-PA/ODN, P-PA/ODN and ODN alone were treated with DNAse I for different time periods and ODN digestion was analyzed with polyacrylamide gel electrophoresis. Reaction mixtures for each experimental group are shown in Table S5. Each sample was treated with DNAse I for 10 min, 30 min, 1 h, 4 h and 24 h at 37 °C. At t = 0, samples had 3 μL of ddH_2_O instead of 3 μL of DNAse I. After the incubation period, samples were loaded onto 10% polyacrylamide gel. Before loading, all samples were incubated with 3 μL of 1% SDS to disrupt electrostatic interaction between ODNs and PAs for 5 min at room temperature. Samples were run for 60 min at 75 V and subsequently 80 min at 50 V (in 1x TAE). All other conditions were identical with the PAGE experiment mentioned above. Band intensities were measured by Image J software. Representative results of three independent experiments are shown.

### Immunizations and determination of antibody responses

Male 10–11 weeks old Balb/c mice were immunized with 500 μL intraperitoneal injections. 6 groups (n = 5) of animals were treated with Ova (antigen) alone, Ova with CpG ODN, Ova with K-PA/CpG ODN or Ova with K-PA/cont. ODN (all in isotonic sucrose solution). 10 μg Ova was given to all animals, while CpG ODN or control ODN amounts were 10 μg in relevant groups. Booster injections were performed at day 15. At days 13 and 28, animals were bled, and sera were obtained. IgG amounts in sera was detected with ELISA. Ova antigen was coated onto 96-well plates, blocked with 1% BSA buffer and serially diluted (10 fold) sera were added onto the wells. IgG was detected with HRP-conjugated anti-IgG. Absorbance in each well was measured after substrate (TMB) addition.

All procedures regarding animals were approved by the Institutional Animal Care and Use Committee of Diskapi Yildirim Beyazit Training and Research Hospital, Ankara, Turkey. This study was carried out in accordance with the approved guidelines.

## Additional Information

**How to cite this article**: Mammadov, R. *et al.* Virus-like nanostructures for tuning immune response. *Sci. Rep.* **5**, 16728; doi: 10.1038/srep16728 (2015).

## Supplementary Material

Supplementary Information

## Figures and Tables

**Figure 1:**
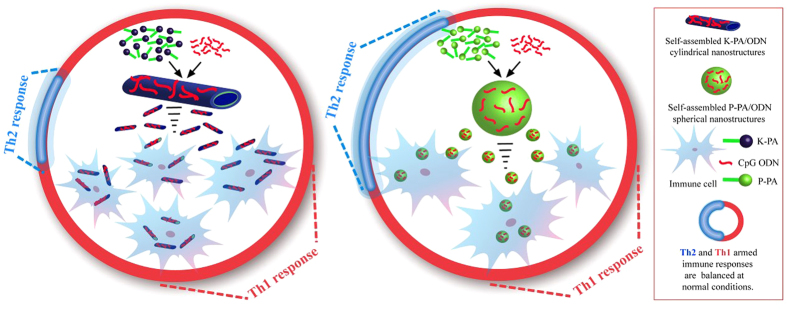
Schematic representation of virus-like nanostructures and tunability of immune response. CpG ODNs mimic immunostimulatory CpG motifs (red) of viral DNA. Mixing CpG ODNs with proline-rich peptides produces nanospheres with 15–20 nm diameter, while mixing with β-sheet forming peptide leads to the formation of one-dimensional nanofibers with 10–15 nm diameter and >200 nm length. CpG ODNs are known to induce the Th1-biased immune response. Delivering them on nanospheres and nanofibers elevates this effect, while nanofiber based delivery is more potent.
