# Supplementary material for: Virus-like nanostructures for tuning immune response
Source: Sci Rep. 2015 Nov 18;5:16728. doi: 10.1038/srep16728 (PMC4649742; doi:10.1038/srep16728)
Supplement: Supplementary Information [file srep16728-s1.docx]

**Supplementary Information for**

**Virus-like nanostructures for tuning immune response**


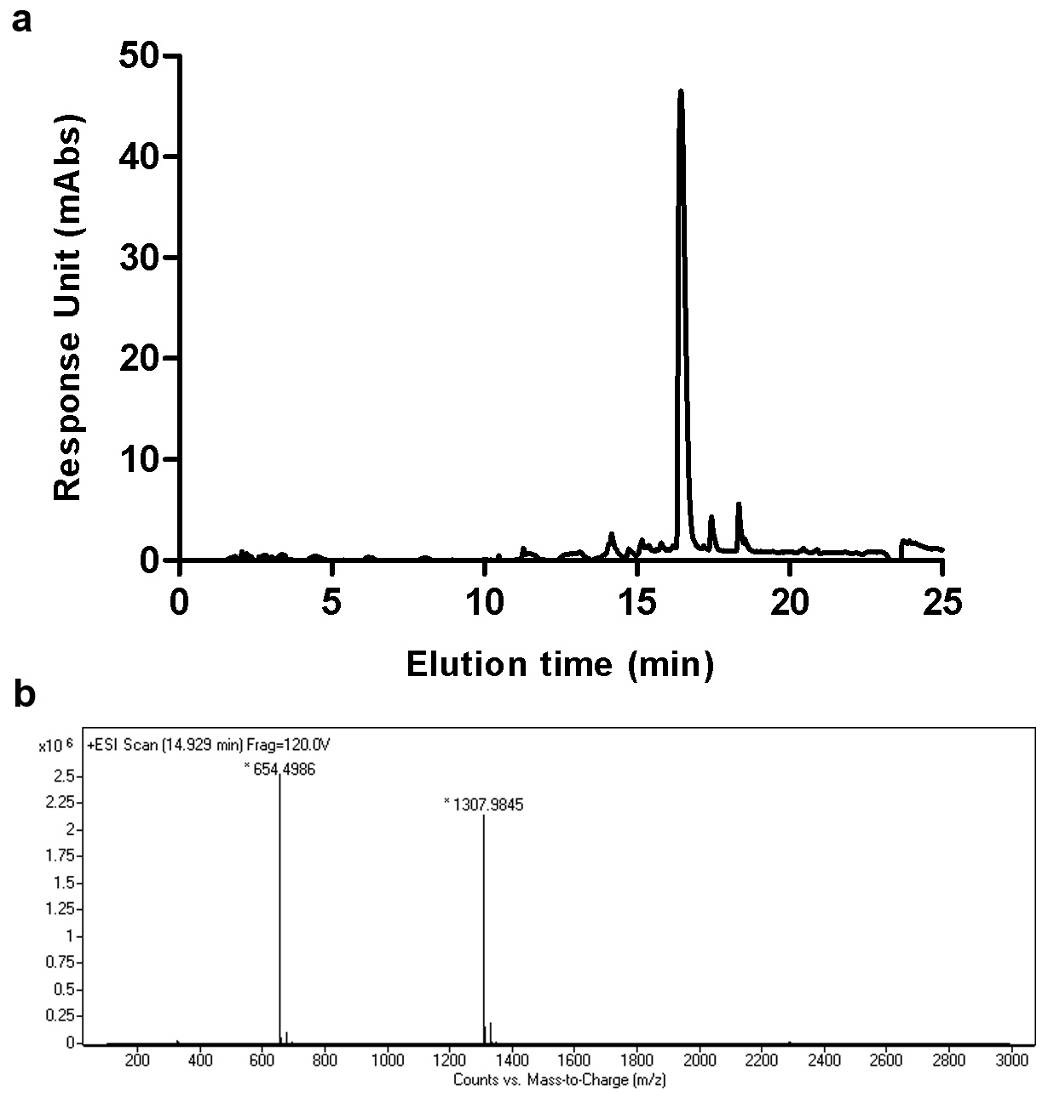


**Figure S1.** (a) HPLC chromatogram of K-PA molecule at 220 nm. (b) Mass spectrometry analysis of K-PA. [M+H]^+^(calculated) = 654.48, [M+H]^+^(observed) = 654.50.


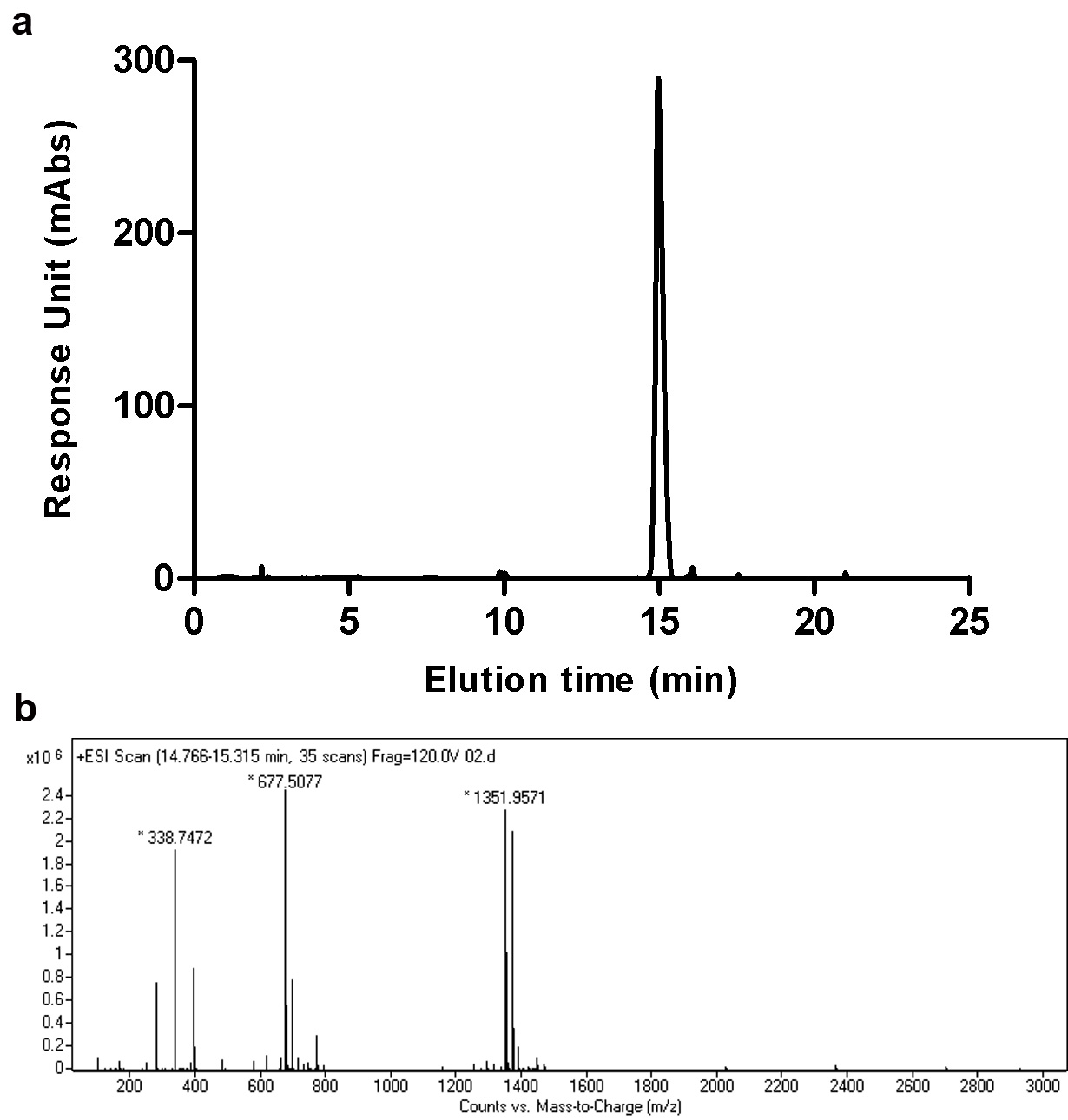


**Figure S2.** (a) HPLC chromatogram of P-PA molecule at 220 nm. (b) Mass spectrometry analysis of P- PA. [M+H]^+^(calculated) = 676.47, [M+H]^+^(observed) = 677.51.

**
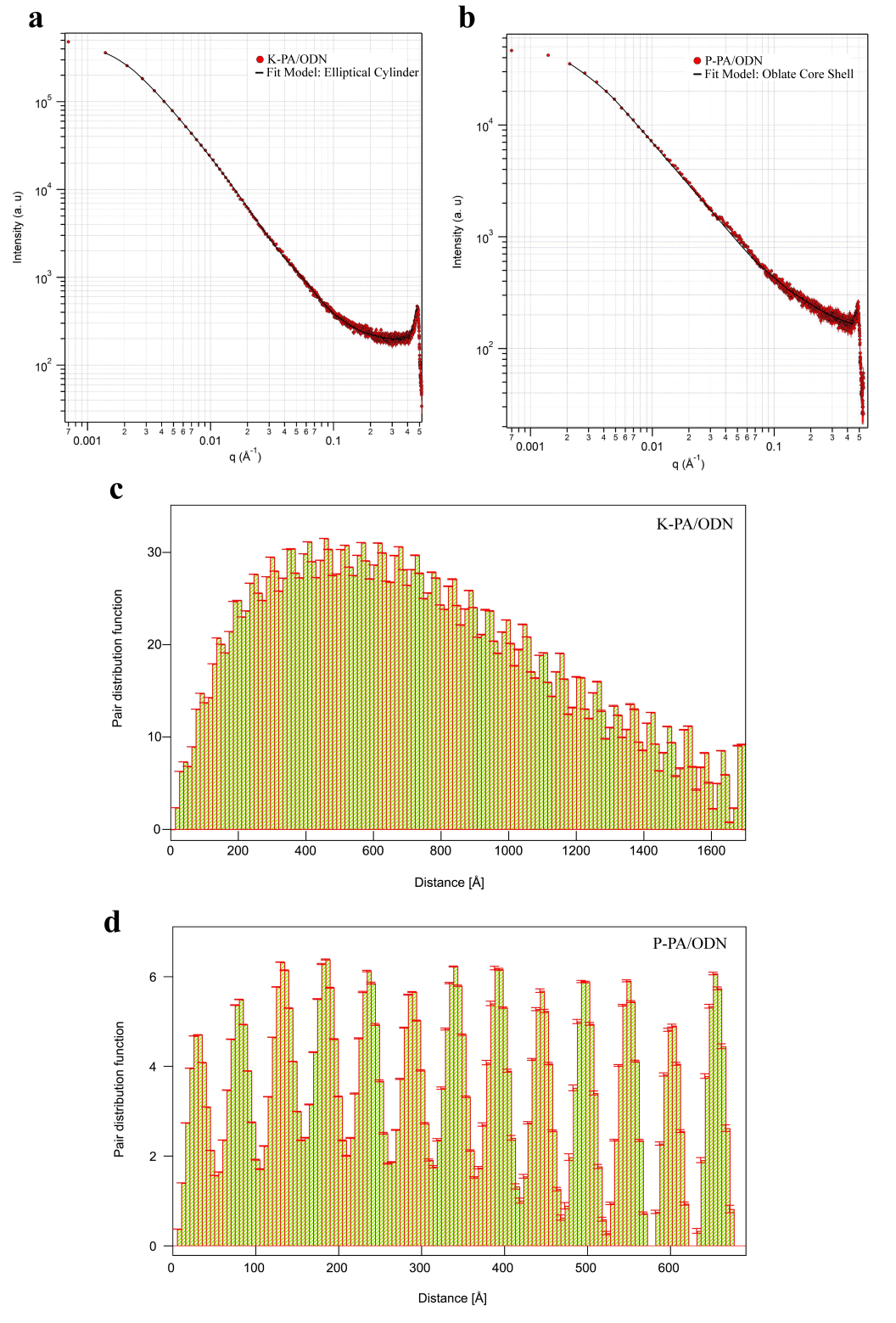
**

**Figure S3. SAXS analyses and model fitting for self-assembled PA/ODN nanostructures.** According to Guinier plots, radius of gyration (R_g_) of (a) K-PA/ODN self-assembled nanostructures was calculated to the best fit as elliptical cylinder model, and (b) P-PA/ODN self-assembled nanostructures was calculated to the best fit as oblate core shell sphere model. PDDF histograms of (c) K-PA/ODN, and (d) P-PA/ODN self-assembled nanostructures also showed characteristics of elliptical cylinder and oblate core shell sphere model, respectively.

**
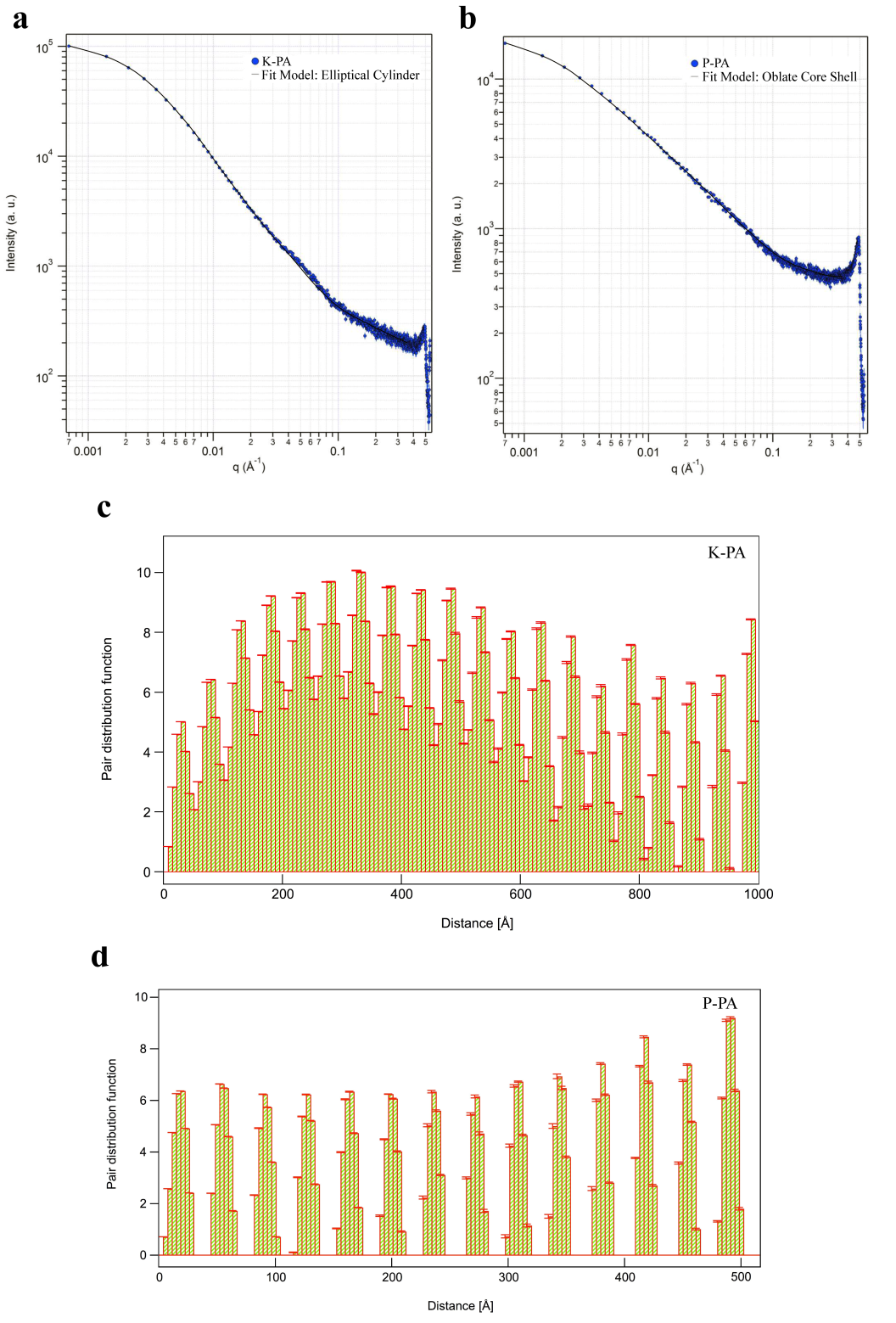
**

**Figure S4. SAXS analyses and model fitting for self-assembled PA nanostructures.** According to Guinier plots, radius gyration (R_g_) of (a) K-PA self-assembled nanostructures was calculated to the best fit as elliptical cylinder model, and (b) P-PA self-assembled nanostructures was calculated to the best fit as oblate core shell sphere model. PDDF histograms of (c) K-PA and (d) P-PA self-assembled nanostructures also showed characteristics of elliptical cylinder and oblate core shell sphere model, respectively.

**Table S1.** Structural results obtained from fits to the SAXS data of K-PA/ODN complexes with elliptical cylinder model.

| ***Fitting Results (Elliptical Cylinder Model)*** | ***K-PA/ODN*** |
| --- | --- |
| **Scale** | 4.6 |
| **Minor radius (nm)** | 7.6±0.3 |
| **Major radius (nm)** | 8.6±0.4 |
| **Length (nm)** | 235.2±1.2 |
| **SLD Cylinder (Å^-2^)** | 6.8×10^-5^ |
| **SLD Solvent (Å^-2^)** | 3.7×10^-6^ |

**Table S2.** Structural results obtained from fits to the SAXS data of P-PA/ODN complexes with oblate core shell sphere model.

| ***Fitting Results (Oblate Core Shell Model)*** | ***P-PA/ODN*** |
| --- | --- |
| **Scale** | 2.6 |
| **Minor core (nm)** | 1.8±0.1 |
| **Minor shell (nm)** | 4.9±0.2 |
| **Major core (nm)** | 2.0±0.1 |
| **Major shell (nm)** | 5.2±0.2 |
| **SLD core (Å^-2^)** | 1.3×10^-6^ |
| **SLD shell (Å^-2^)** | 3.1×10^-5^ |
| **SLD Solvent (Å^-2^)** | 1.4×10^-7^ |

**Table S3.** Structural results obtained from fits to the SAXS data of K-PA with elliptical cylinder model.

| ***Fitting Results (Elliptical Cylinder Model)*** | ***K-PA*** |
| --- | --- |
| **Scale** | 4.6 |
| **Minor radius (nm)** | 7.0±0.3 |
| **Major radius (nm)** | 8.0±0.3 |
| **Length (nm)** | 235.2±1.2 |
| **SLD Cylinder (Å^-2^)** | 4.8×10^-5^ |
| **SLD Solvent (Å^-2^)** | 3.7×10^-6^ |

**Table S4.** Structural results obtained from fits to the SAXS data of P-PA with oblate core shell sphere model.

| ***Fitting Results (Oblate Core Shell Model)*** | ***P-PA*** |
| --- | --- |
| **Scale** | 2.6 |
| **Minor core (nm)** | 1.2±0.1 |
| **Minor shell (nm)** | 3.2±0.2 |
| **Major core (nm)** | 1.5±0.1 |
| **Major shell (nm)** | 3.6±0.2 |
| **SLD core (Å^-2^)** | 1.3×10^-6^ |
| **SLD shell (Å^-2^)** | 2.0×10^-5^ |
| **SLD Solvent (Å^-2^)** | 1.4×10^-7^ |

**
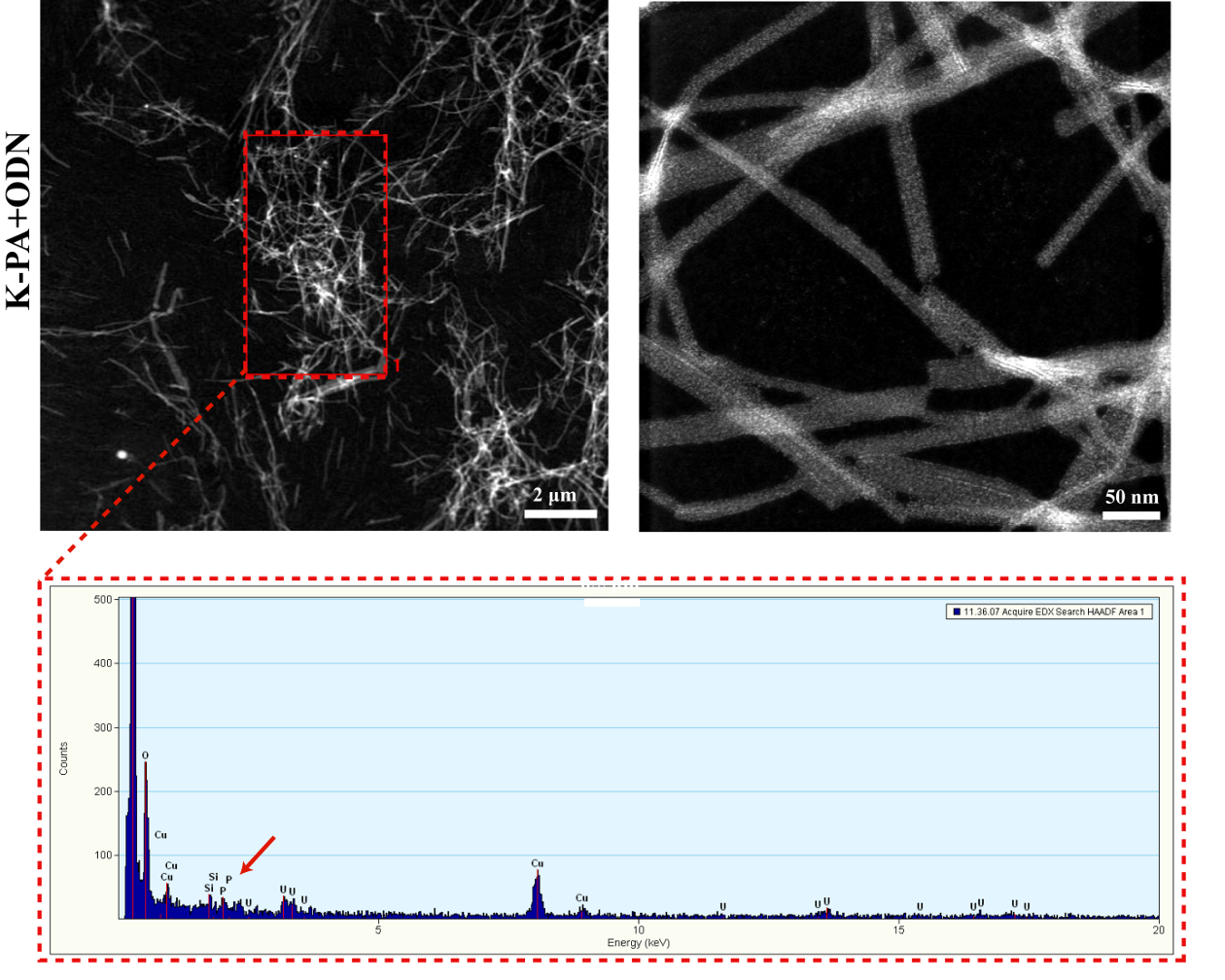
**

**Figure S5. STEM images of self-assembled K-PA/ODN peptide fibers.** The initial fiber formation and peptide bundles can be seen in images (scale bars: left image 2 µm, right image 50 nm). EDX analysis (from the region shown by the red rectangle in left STEM image revealed P atoms on the peptide bundles and self-assembly of DNA molecules with K-PA.

**
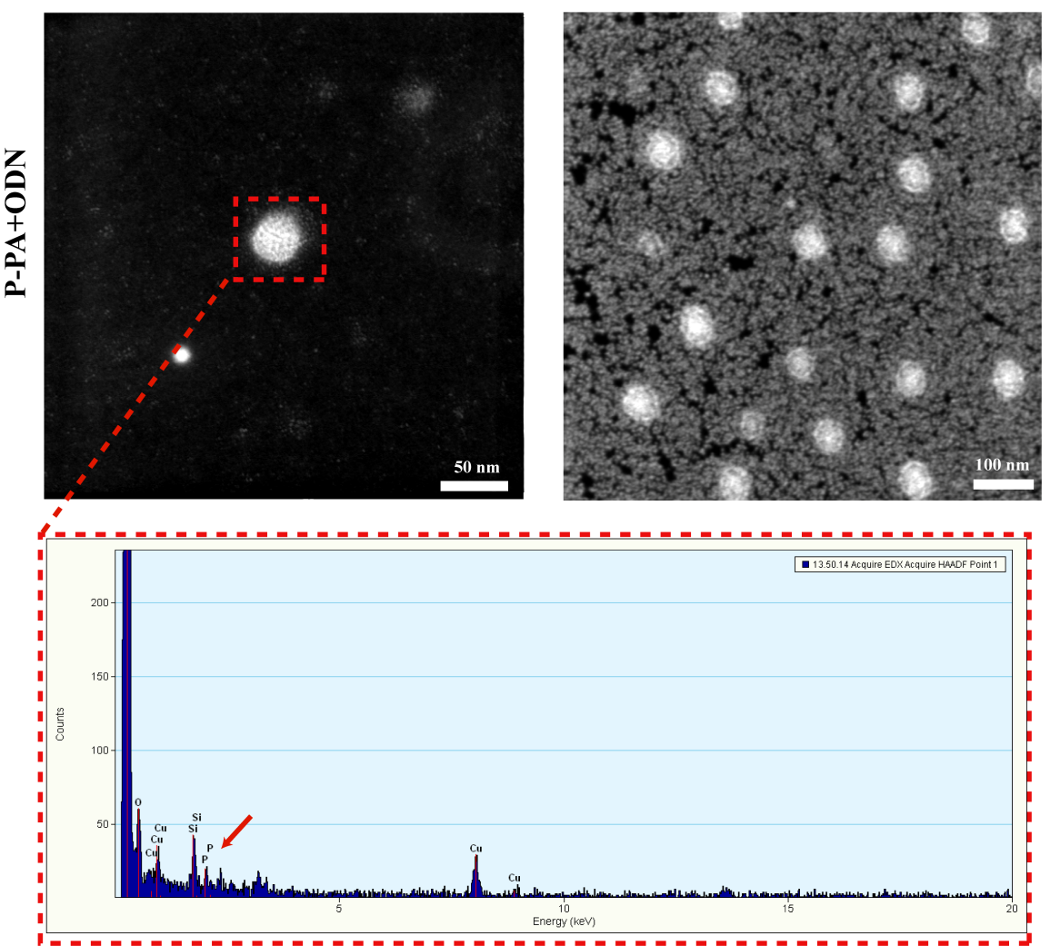
**

**Figure S6. STEM images of self-assembled P-PA/ODN peptide spherical nanostructures.** The spherical morphology of the nanostructures can be seen in images (scale bars: left image 50 nm, right image 100 nm). EDX analysis (from the region shown by the red rectangle in left STEM image revealed P atoms on the spherical nanostructures and self-assembly of DNA molecules with P-PA.

**
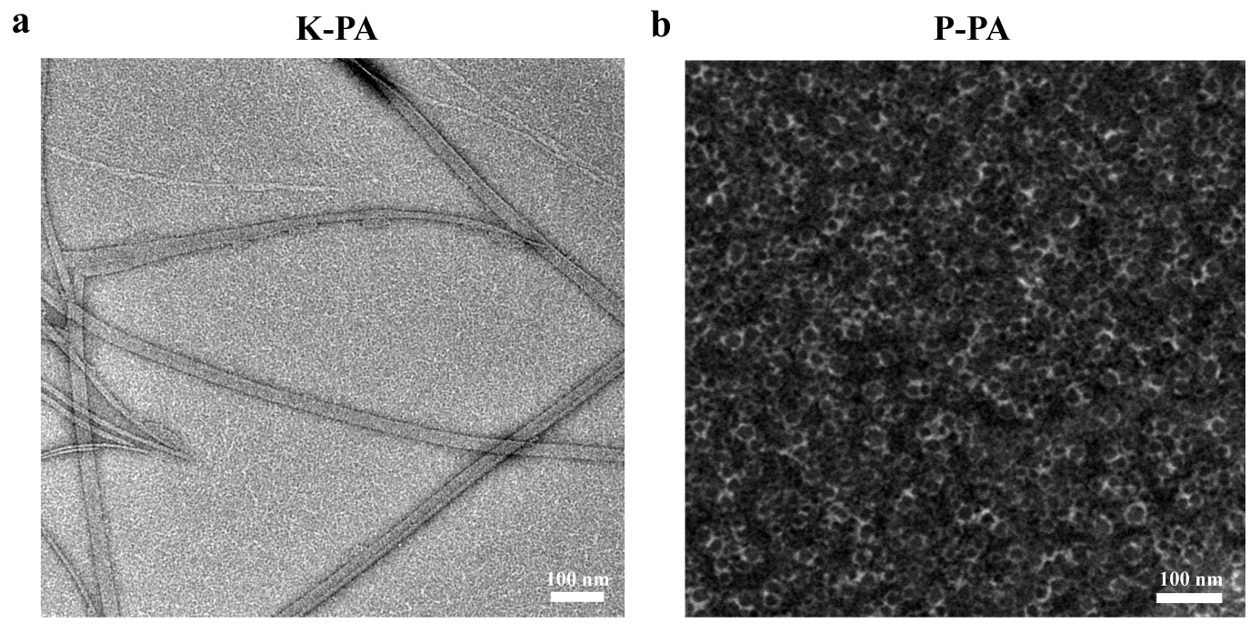
**

**Figure S7. TEM and STEM images of self-assembled K-PA and P-PA nanostructures.** (a) The K-PA fiber formation and peptide bundles can be seen in images. (b) The spherical morphology of the P-PA self-assembled nanostructures can be seen in images (scale bars: 100 nm).

**
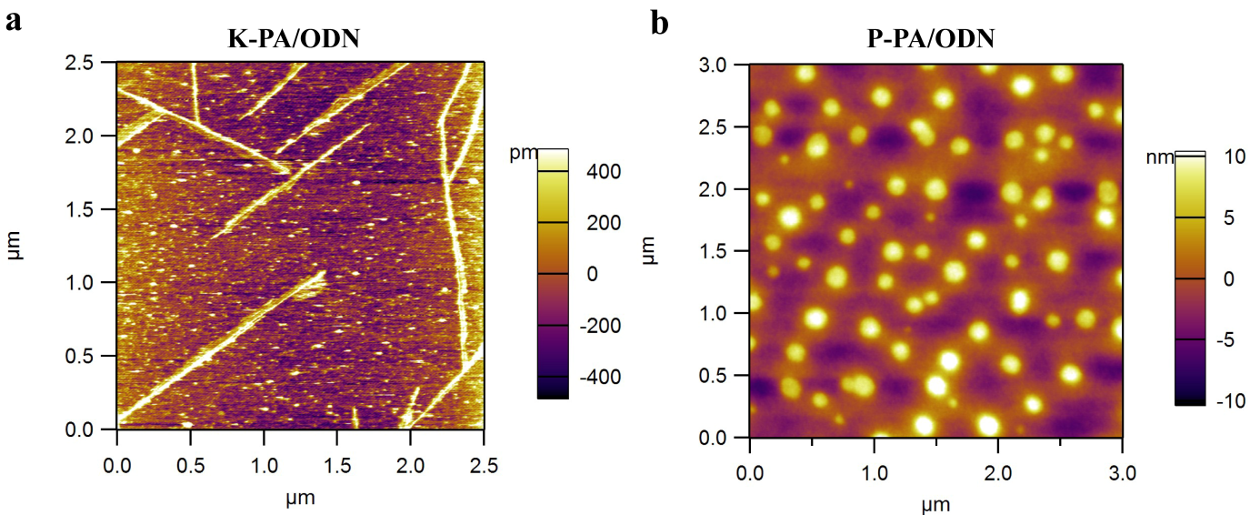
**

**Figure S8. AFM images of self-assembled K-PA/ODN and P-PA/ODN nanostructures.** (a) K-PA/ODN solution that was dried overnight. (b) P-PA/ODN solution that was dried overnight. The imaging was performed in contact mode.


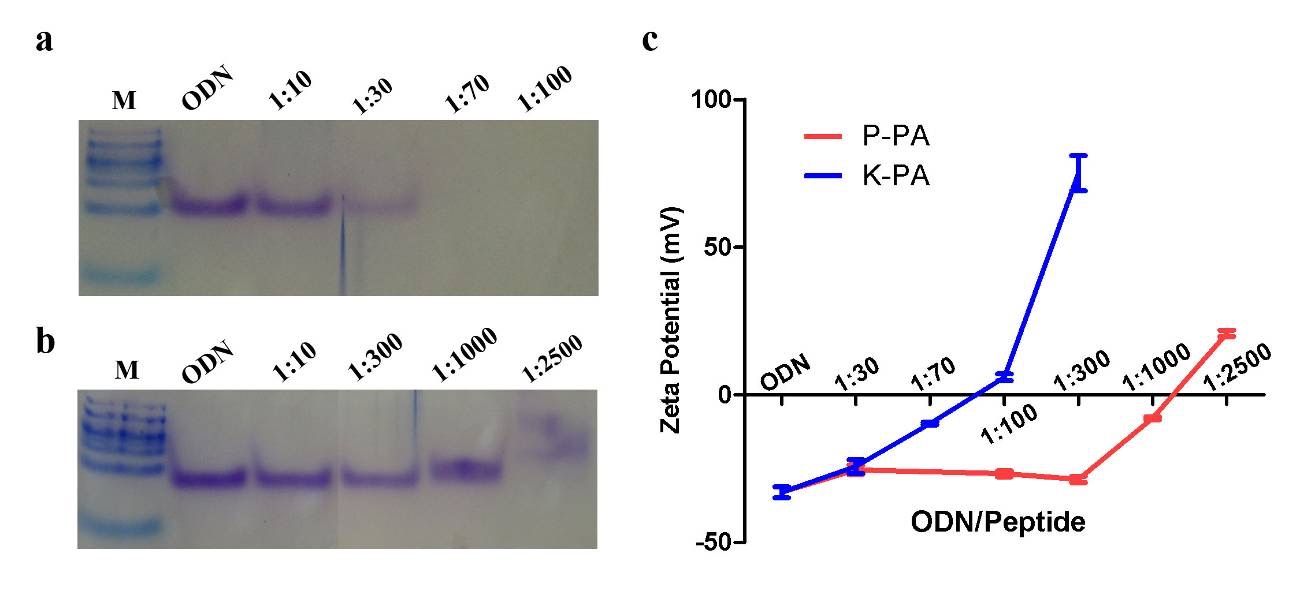


**Figure S9. Determination of critical ODN/Peptide ratio.** Peptide/ODN formulations with varying ratios for (a) K-PA, and (b) P-PA were run on PAGE. ODN concentration was kept constant; the peptide concentrations were adjusted to make all ODNs in solution to bind to nanostructures. (c) Zeta potential measurements of these formulations.

**
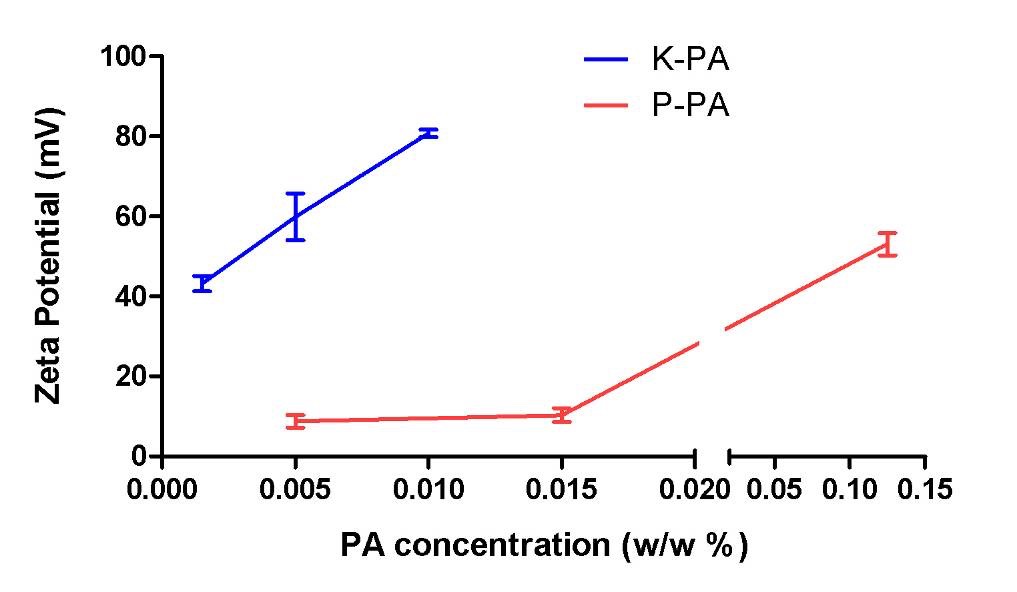
**

Figure S10. Zeta potential values of PA molecules at various concentrations. K-PA shows higher zeta potential values than P-PA at similar concentrations.


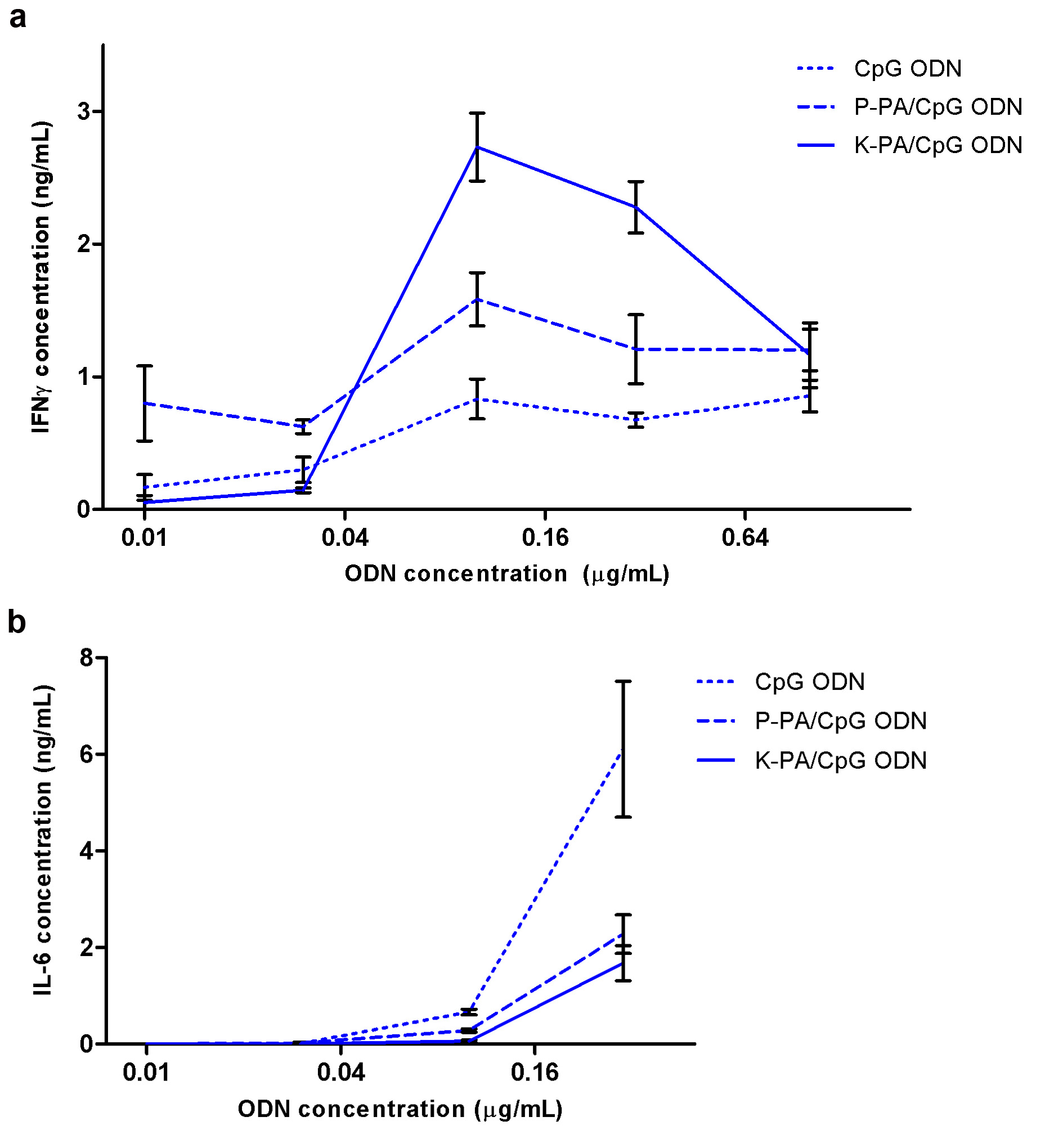


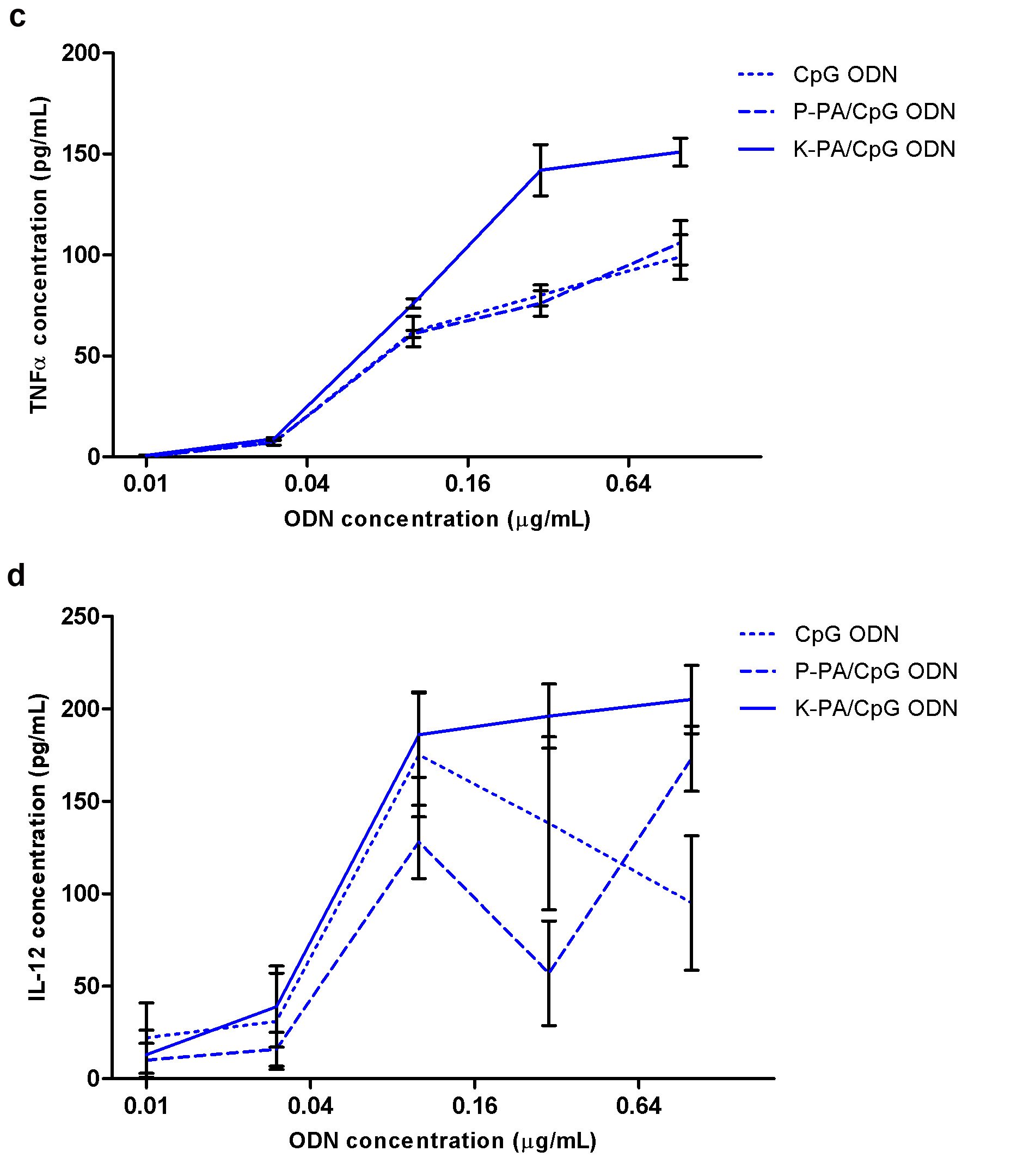


**Figure S11. Effect of nanostructures on cytokine production of splenocytes induced by dose-dependent CpG ODN treatment**. After treating splenocytes with ODN alone or bound to nanostructures, (a) IFNγ, (b) IL-6, (c) TNFα, (d) IL-12 cytokines secreted to culture media were detected by ELISA.

**
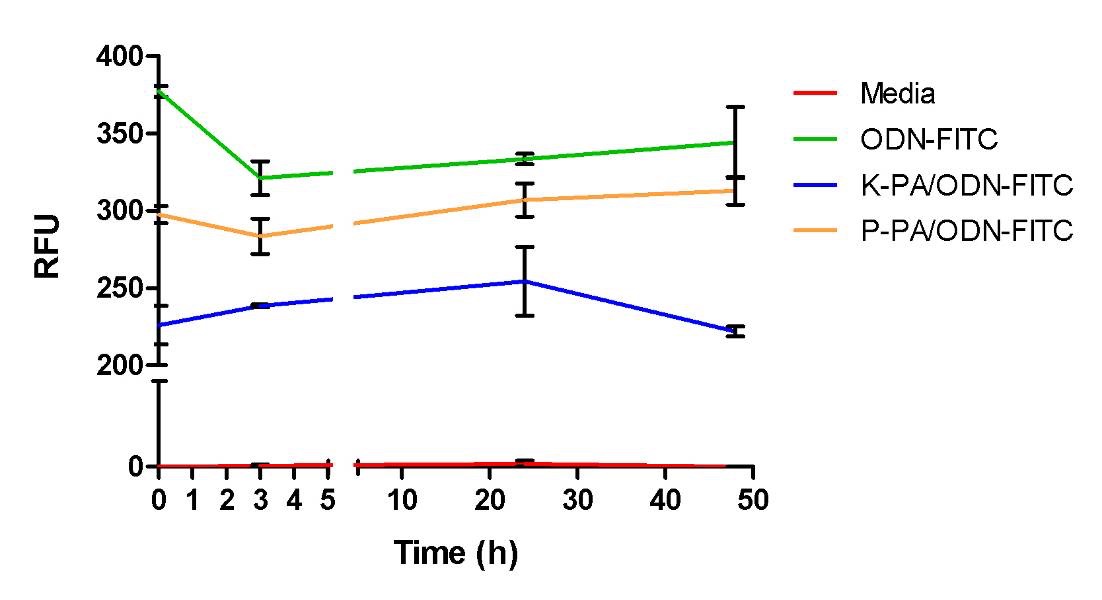
**

**Figure S12. Stability of peptide-ODN nanostructures in cell culture media.** FITC-tagged ODNs were incubated either alone or in complex with spherical or fibrous nanostructures in cell culture media for 48 hours. Relative fluorescence unit (RFU) was measured at various time points.


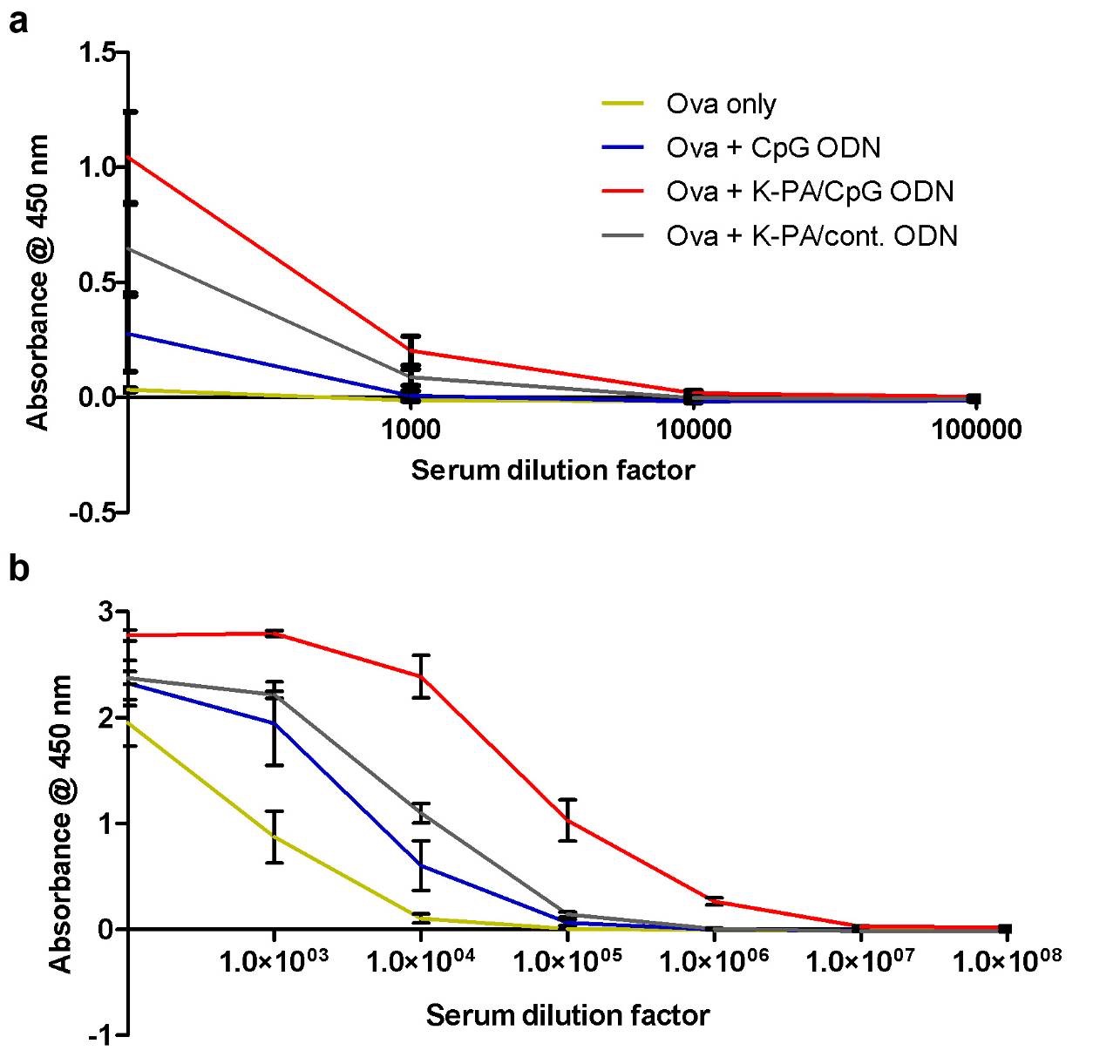


**Figure S13.** Serum titration curves for Ova-specific IgG signal. (a) Day 13 sera after primary injection. (b) Day 28 sera (13 days after secondary injection).


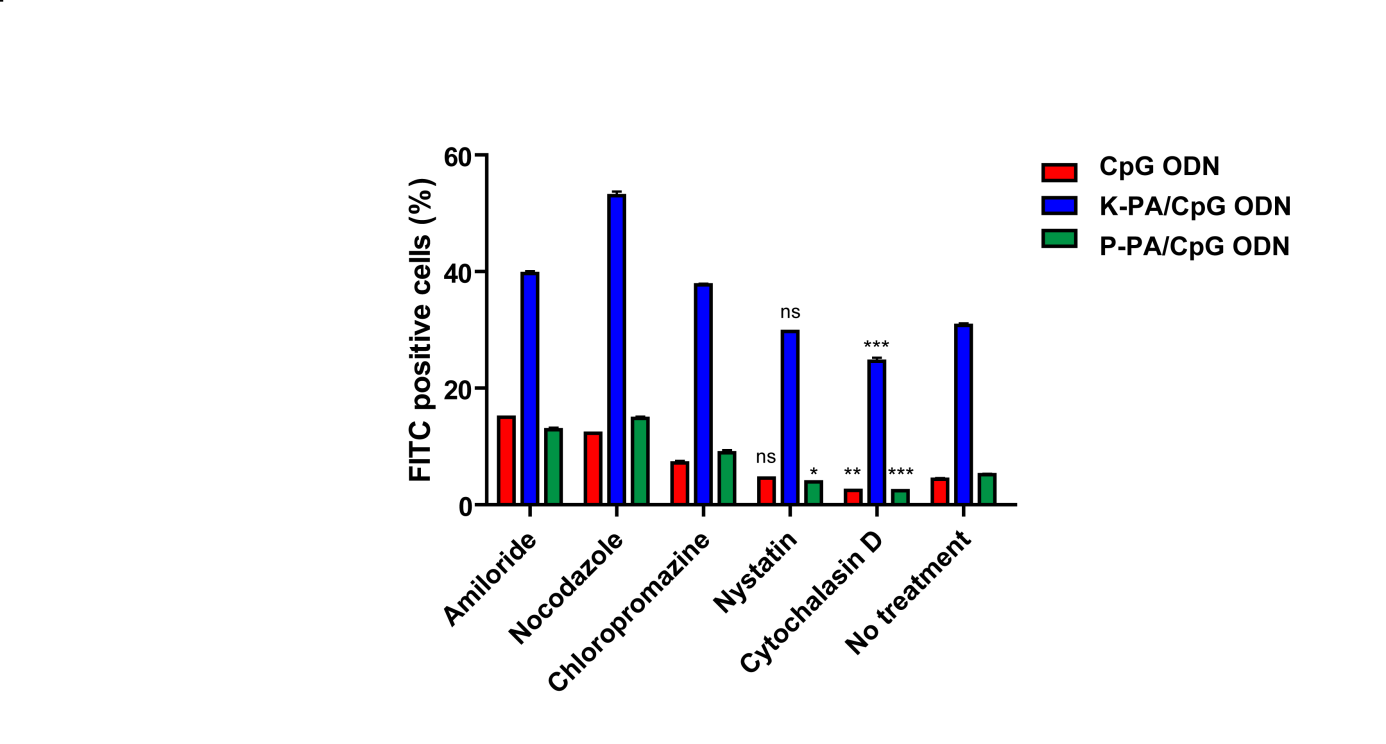


**Figure S14.** Internalization profiles of FITC-ODN alone or bound with nanostructures into macrophage cells after the inhibition of specific internalization pathways using chemical inhibitors at 2 h. Red, blue, and green bars indicate only CpG ODN, K-PA/CpG ODN, and P-PA/CpG ODN, respectively. (*p<0.05, **p<0.01 , ***p<0.001 according to two-way ANOVA)


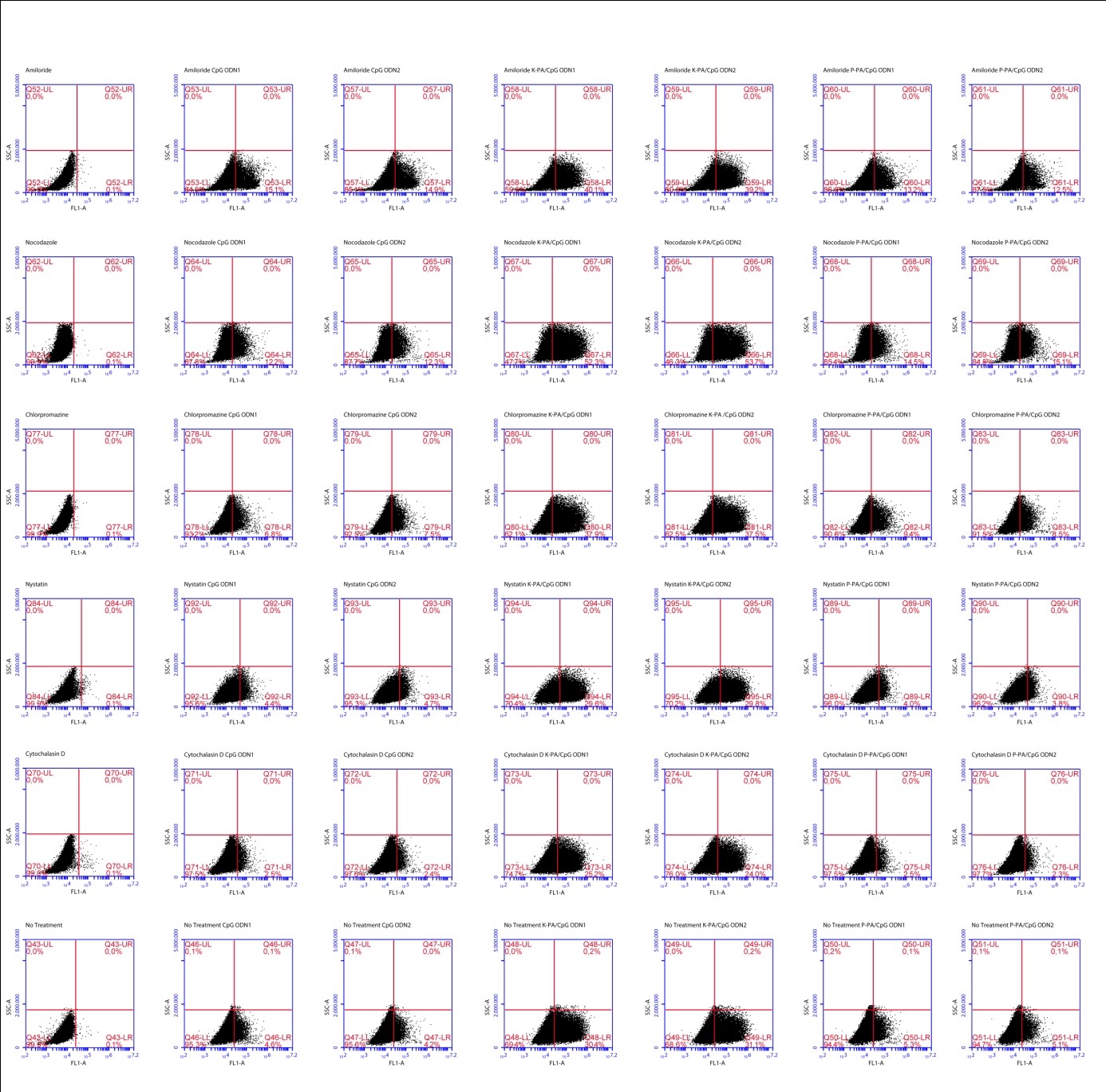


**Figure S15.** FACS plots of the cells gated by SSC (side scatter channel) and FSC (forward scatter channel) using non-treated control.


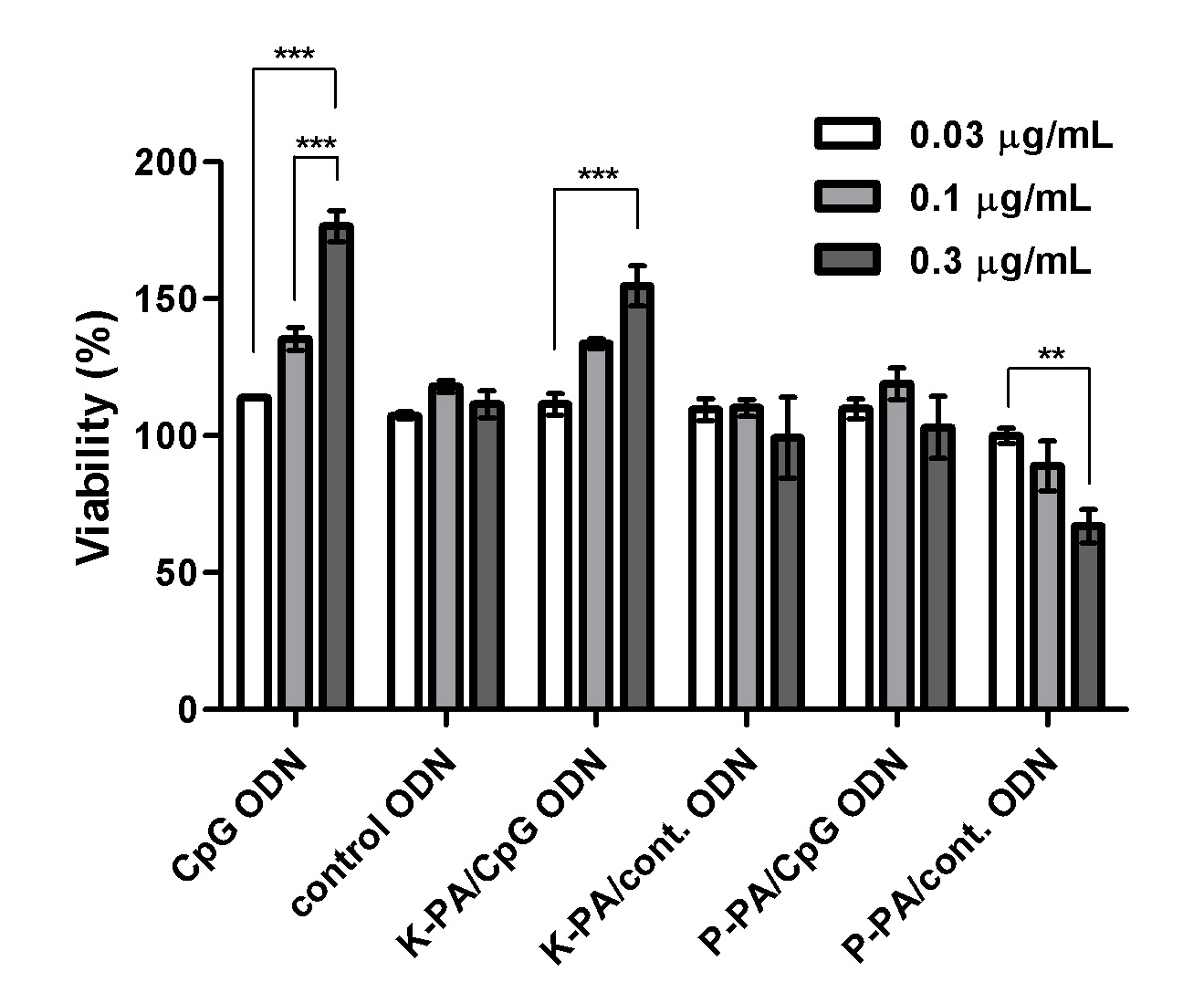


**Figure S16.** Evaluation of viability of splenocytes treated with ODN and their complexes with nanofibers and nanospheres via MTT assay. Statistical analysis was performed by using two-way ANOVA with Bonferroni post-tests (***p<0.001, **p<0.01).

**Table S5.** Reaction mixtures for DNAse I treatment.

| Experimental groups | | |
| --- | --- | --- |
| Only ODN | K-PA/ODN | P-PA/ODN |
| 12 µL, 25 µg/mL ODN | 12 µL, 25 ng/mL ODN | 7.5 µL, 40 µg/mL ODN |
| 12 µL, ddH_2_O | 12 µL, 0.025% K-PA | 7.5 µL, 1% P-PA |
|  |  | 9 µL, ddH_2_O |
| 3 µL, DNAse Buffer | 3 µL, DNAse Buffer | 3 µL, DNAse Buffer |
| 3 µL, DNAse I | 3 µL, DNAse I | 3 µL, DNAse I |

**Table S6.** Chemical inhibitors used in this paper.

| **Inhibitor** | **Mechanism** | **Affected pathway** |
| --- | --- | --- |
| **Amiloride** | Na+/H+ exchanger. Unspecific. | macropinocytosis |
| **Nocodazole** | Microtubule depolymerization and alteration mitosis | mitosis |
| **Chlorpromazine** | clathrin: alteration of clathrin-coat assembly, membrane fluidity and permeability | clathrin dependent endocytosis |
| **Nystatin** | Binding to plasma membrane cholesterol and alteration of caveolae dependent endocytosis | caveolae dependent endocytosis |
| **Cytochalasin D** | actin: blockage of its polymerization avoiding microfilaments action and inhibition of other endocytosis routes. | macropinocytosis |
